# Supplementary figures and images for: Cohort profile: the United Kingdom Childhood Cancer Study (UKCCS) – a UK-wide population-based study examining the health of cancer survivors
Source: BMJ Open. 2023 Nov 17;13(11):e073712. doi: 10.1136/bmjopen-2023-073712 (PMC10660444; doi:10.1136/bmjopen-2023-073712)

**Supplementary Figure 1****United Kingdom Childhood Cancer Study (UKCCS) administrative areas**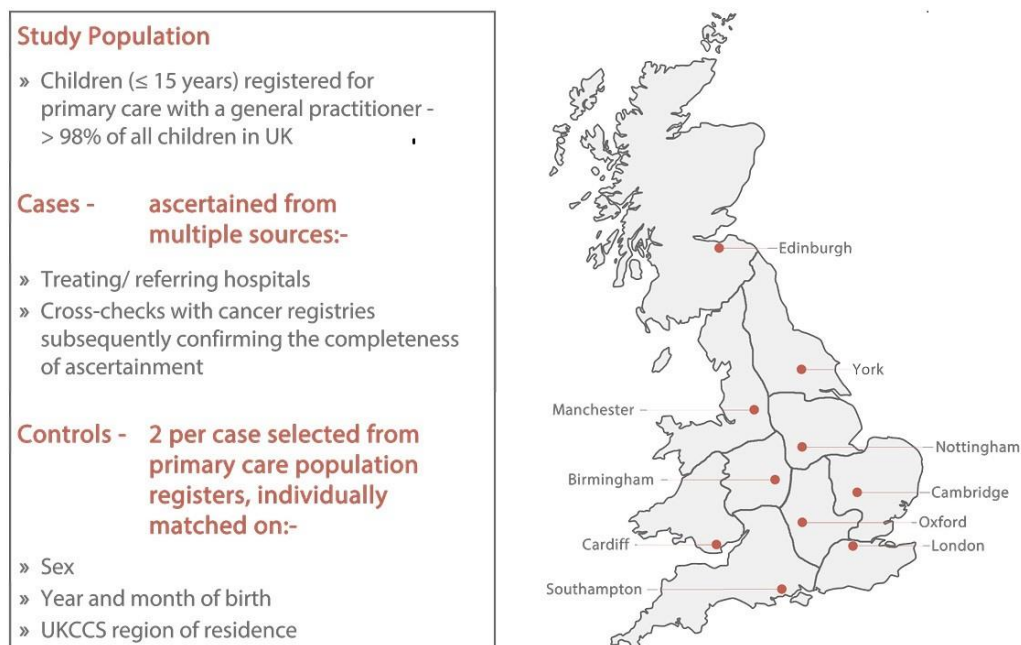

Supplement: Supplementary data [file bmjopen-2023-073712supp001.pdf]
