# Supplementary material for: Cohort profile: the United Kingdom Childhood Cancer Study (UKCCS) – a UK-wide population-based study examining the health of cancer survivors
Source: BMJ Open. 2023 Nov 17;13(11):e073712. doi: 10.1136/bmjopen-2023-073712 (PMC10660444; doi:10.1136/bmjopen-2023-073712)
Supplement: Supplementary data [file bmjopen-2023-073712supp002.pdf]

Supplementary Figure 2

United Kingdom Childhood Cancer Study (UKCCS) linkage to national administrative datasets

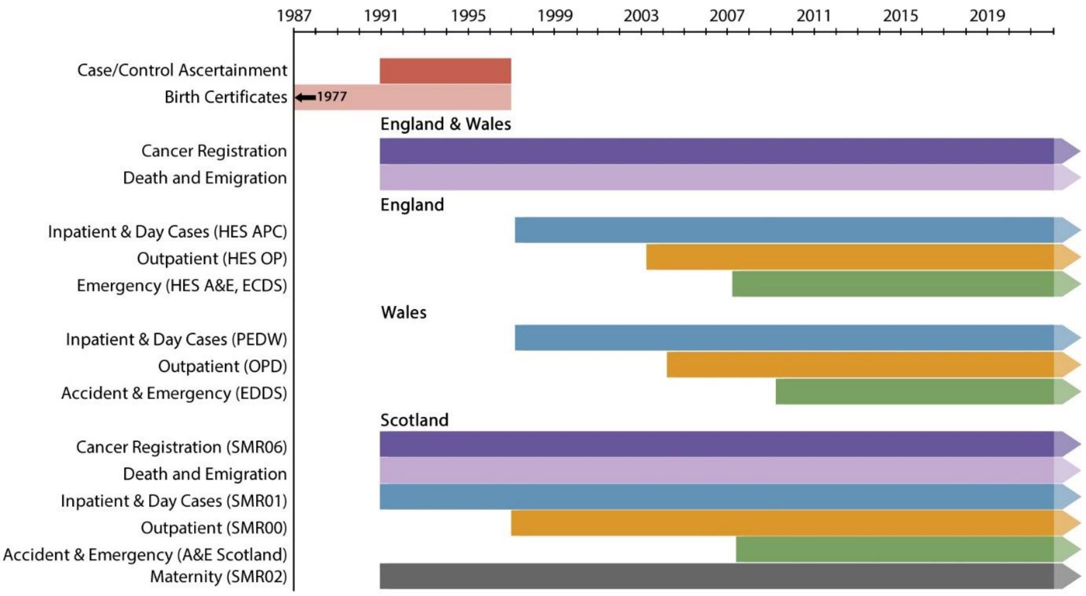

UKCCS: United Kingdom Childhood Cancer Study; HES: Hospital Episode Statistics; APC: Admitted Patient Care; OP: Outpatients; ECDS: Emergency Care Dataset; PEDW: Patient Episode Data for Wales; OPD: Outpatient Data (Wales); EDD: Emergency Department Dataset (Wales); SMR: Scottish Morbidity Record
