# Supplementary material for: Cohort profile: the United Kingdom Childhood Cancer Study (UKCCS) – a UK-wide population-based study examining the health of cancer survivors
Source: BMJ Open. 2023 Nov 17;13(11):e073712. doi: 10.1136/bmjopen-2023-073712 (PMC10660444; doi:10.1136/bmjopen-2023-073712)
Supplement: Author's manuscript [file bmjopen-2023-073712.draft_revisions.pdf]

BMJ Open

BMJ Open is committed to open peer review. As part of this commitment we make the peer review history of every article we publish publicly available.

When an article is published we post the peer reviewers' comments and the authors' responses online. We also post the versions of the paper that were used during peer review. These are the versions that the peer review comments apply to.

The versions of the paper that follow are the versions that were submitted during the peer review process. They are not the versions of record or the final published versions. They should not be cited or distributed as the published version of this manuscript.

BMJ Open is an open access journal and the full, final, typeset and author-corrected version of record of the manuscript is available on our site with no access controls, subscription charges or pay-per-view fees (http://bmjopen.bmj.com).

If you have any questions on BMJ Open's open peer review process please email info.bmjopen@bmj.com

BMJ Open

Cohort Profile: The United Kingdom Childhood Cancer Study (UKCCS): a UK-wide population-based study examining the health of cancer survivors

Journal:	*BMJ Open*
Manuscript ID	bmjopen-2023-073712
Article Type:	Cohort profile
Date Submitted by the Author:	14-Mar-2023
Complete List of Authors:	Roman, Eve; University of York, Health Sciences<br>Kane, Eleanor; University of York, Health Sciences<br>Smith, Alexandra; University of York, Health Sciences<br>Howell, Debra; University of York, Health Sciences<br>Sheridan, Rebecca; University of York,<br>Simpson, Jill; University of York<br>Bonaventure, Audrey; INSERM<br>Kinsey, Sally; Leeds Children's Hospital, Paediatric Haematology
Keywords:	Leukaemia < HAEMATOLOGY, Lymphoma < ONCOLOGY, EPIDEMIOLOGY, PUBLIC HEALTH, Quality of Life, Risk Factors

SCHOLARONE™
Manuscripts

I, the Submitting Author has the right to grant and does grant on behalf of all authors of the Work (as defined in the below author licence), an exclusive licence and/or a non-exclusive licence for contributions from authors who are: i) UK Crown employees; ii) where BMJ has agreed a CC-BY licence shall apply, and/or iii) in accordance with the terms applicable for US Federal Government officers or employees acting as part of their official duties; on a worldwide, perpetual, irrevocable, royalty-free basis to BMJ Publishing Group Ltd ("BMJ") its licensees and where the relevant Journal is co-owned by BMJ to the co-owners of the Journal, to publish the Work in this journal and any other BMJ products and to exploit all rights, as set out in our licence.

The Submitting Author accepts and understands that any supply made under these terms is made by BMJ to the Submitting Author unless you are acting as an employee on behalf of your employer or a postgraduate student of an affiliated institution which is paying any applicable article publishing charge ("APC") for Open Access articles. Where the Submitting Author wishes to make the Work available on an Open Access basis (and intends to pay the relevant APC), the terms of reuse of such Open Access shall be governed by a Creative Commons licence – details of these licences and which Creative Commons licence will apply to this Work are set out in our licence referred to above.

Other than as permitted in any relevant BMJ Author's Self Archiving Policies, I confirm this Work has not been accepted for publication elsewhere, is not being considered for publication elsewhere and does not duplicate material already published. I confirm all authors consent to publication of this Work and authorise the granting of this licence.

For peer review only

Cohort Profile: The United Kingdom Childhood Cancer Study (UKCCS): a UK-wide population-based study examining the health of cancer survivors

Eve Roman[1*]

Eleanor Kane[1]

Alexandra Smith[1]

Debra Howell[1]

Rebecca Sheridan [1]

Jill Simpson[1]

Audrey Bonaventure[2]

Sally Kinsey[1]

[1] Epidemiology and Cancer Statistics Group, Department of Health Sciences, University of York, YO10 5DD, UK

*Corresponding author: eve.roman@york.ac.uk

Word count: Main text = 2471

For peer review only - http://bmjopen.bmj.com/site/about/guidelines.xhtml

For peer review only

Abstract (300)

Purpose: The UKCCS matched-cohort was established to examine the longer-term morbidity and mortality of individuals previously diagnosed with cancer before 15 years of age; comparing the healthcare patterns observed in 5-year cancer survivors to baseline activity seen in age- and sex-matched individuals from the general population.

Participants: Predicated on a national childhood cancer case-control study conducted in the early 1990s (4430 cases and 9758 controls) in England, Scotland and Wales, the case-cohort comprises 3125 cancer survivors (>5 years), and the comparison-cohort 7161 age- and sex- matched individuals from the general population who did not have cancer as a child. Participants are being followed-up via linkages to national administrative healthcare databases (deaths, cancers, and secondary-care hospital activity) that are updated annually.

Findings to date: Enabling the creation of matched cohorts with minimal selection bias and loss to follow-up, the original case-control study registered all newly diagnosed cases of childhood cancer and their corresponding controls, regardless of their family's participation. Early findings based on the case-cohort alone highlighted survival variations with age and sex across subtypes, as well as with deprivation among survivors of acute lymphoblastic leukaemia. More recently, comparing healthcare patterns in the case-cohort to those in the comparison-cohort over a 25-year period, we showed that adding to their excess risks of death and cancer, 5-year survivors of childhood ALL experienced excess outpatient and inpatient activity across their TYA years; which was not related to routine follow-up monitoring. Involving most clinical specialties, associations were striking; showing no signs of diminishing over time.

Future plans: The UKCCS's maturing cohorts provide the foundation for tracking the health of childhood cancer survivors through their lifetimes. This will include: examining associations with deprivation; patterns of healthcare among survivors of rarer less well-studied childhood cancers; and whether mortality and morbidity patterns ever return to general population levels.

Strengths and limitations of this study (max 5 bullet points)

- Covering England, Scotland and Wales, findings from this national study are based on routinely compiled linked health data, not self-report
- Regardless of whether or not their families participated in the original case-control study, all children are included in the matched cohort, enabling the investigation of selection bias.
- With minimal selection bias and loss to follow-up, the individually age- and sex-matched comparison cohort has a similar deprivation distribution to the case-cohort, providing a robust baseline against which to evaluate the impact of deprivation across the life-course
- Analyses are constrained by the fact that national administrative healthcare data are primarily collected for administrative and clinical purposes, and not for research.
- With respect to weaknesses, lack of primary care data and information from psychosocial services are obvious deficiencies that currently affects all UK record-linkage studies of the type described here.

For peer review only - http://bmjopen.bmj.com/site/about/guidelines.xhtml

INTRODUCTION

In recent decades, advances in molecular biology and therapy have transformed the landscape for several childhood cancers, changing many from rapidly fatal diseases to treatable conditions with a good prognosis; overall survival now exceeds 80% in the UK and other economically developed countries[1,2]. Accordingly, it is estimated that by 2030 there will be around three-quarters of a million childhood cancer survivors in Europe alone; this number increasing as therapies improves and populations age[3]. Whilst the majority of cancers in children are more responsive to chemotherapy than those in adults, treatments are often more aggressive, and adverse health problems are well-known to occur later in life (e.g. second cancers, cardiac and bone problems, and fertility issues)[4–7].

The national cohort described in this report was established to examine the relationship between cancers diagnosed before 15 years of age and other morbidities and health-states in the years after childhood cancer diagnosis. It is predicated on the structures established in the United Kingdom Childhood Cancer Study (UKCCS), which was set-up as a national population-based case-control study in the early 1990s in England, Scotland and Wales to investigate a wide range of possible cancer causes[8]. The children in the original UKCCS are currently aged between 26-45 years, and the UKCCS design has been transformed into a matched cohort, with all cases and controls (regardless of parents' participation in the original case-control study) being tracked forwards in time via annually updated linkages to national administrative healthcare databases (mortality, cancers, and hospital secondary care)[9–11]. This report describes the underpinning methods and summarises some initial findings.

COHORT DESCRIPTION

Full details of the UKCCS case-control study (ukccs.org) which provides the foundation of the matched-cohort described here has been published[8,12,13]. Briefly, the sampling frame for cases and controls comprised all children registered with the National Health Service (NHS) in England, Scotland or Wales at the time the study was conducted (>98% of the childhood population). Overseen by a management committee that included epidemiologists, statisticians, paediatric oncologist/haematologists and expert scientists that was chaired by Professor Sir Richard Doll, 10 UKCCS administrative areas were created for the purposes of data collection. Each of these areas was the responsibility of a well-established epidemiological research centre[8].

The study began on 01/01/1991 in Scotland, and either 01/04/1992 or 01/09/1992 in the nine UKCCS administrative areas of England and Wales. In Scotland, case accrual ended on 31/12/1994, and in England and Wales, it was restricted to children diagnosed with leukaemia or non-Hodgkin lymphoma throughout 1995, and leukaemia alone throughout 1996. In order to ensure ascertainment completeness, proactive cancer notification systems were established in all diagnostic/treatment centres across the UK; the majority of cases being notified at the point of diagnosis directly to the relevant UKCCS administrative centre by paediatric oncologists or haematologists practising in the study area. Subsequent crosschecks were made against regional and national population-based cancer registries[8].

With the permission of the child's clinical team, the parents of children with cancer were subsequently contacted. Each child whose parents agreed to be interviewed (3835/4430; 87%) was individually matched on sex, date of birth (month and year) and UKCCS region of residence to 10 controls that were randomly selected from the same population-based health service authority list as the case. With their general practitioner's (GP's) permission, the parents of the first two controls on the list ("first-choice" controls) were contacted and asked to participate in the study; but if the GP declined, or the parents did not wish to be interviewed, the next child was selected, and this procedure was repeated until two control families agreed to take part.

For peer review only - http://bmjopen.bmj.com/site/about/guidelines.xhtml

In order to monitor the characteristics of respondents and non-respondents across the study, and examine the potential impact of selection/participation bias, the registration details of all cases and controls were retained, regardless of whether or not their families were interviewed. Using standard methods, address postcodes were used to allocate deprivation scores for the home at diagnosis (all registered cases) or pseudo-diagnosis (all registered controls; date coinciding with the exact age that the corresponding case was diagnosed); and also at the time they were born (using mother's address on the child's birth certificate). Full details of the methods used have been previously published [8,12]. Briefly, deprivation categories were then derived by dividing the continuous score for the national 1991 census enumeration areas into five equally-sized groups, with group one representing the most affluent and group five the most deprived.

Illustrating the UKCCS's potential to examine issues relating to selection bias, **Table 1** distributes all registered cases and controls according to their participation status and area-based deprivation of their home at diagnosis (cases) or pseudo-diagnosis (controls; date coinciding with the exact age that the corresponding case was diagnosed). With roughly 20% of the population in each quintile, the deprivation distribution of all 4430 cases and the 7663 "first-choice" controls targeted for interview are broadly similar. However, as expected, both the 595 (10.8%) cases and 2137 (27.9%) controls whose parents were not interviewed were more likely to have lived in deprived areas; the effect being most pronounced in the control population with 32.7% residing in the most deprived quintile. Hence, exacerbated by the fact that participation among case families was higher (3835/4430=86.6%) than among control families (5526/7663=72.1%), the interviewed case and first-choice control distributions differed significantly from each other (χ^2=15.1, p=0.004). Interestingly, the deprivation distribution of participating first-choice controls (n=5526) and replacement controls (n=2095) are broadly similar (χ^2=6.98, p=0.14).

For peer review only

Table 1: Numbers (%) of UKCCS cases and controls distributed by interview status and area-based deprivation at diagnosis

	**Targeted for interview**		**Parents did not participate**		**Parents participated**		
	All diagnosed cases	All first-choice controls[a]	Cases	First-choice controls	Cases	First-choice controls	Replacement controls
	**N (%)**	**N (%)**	**N (%)**	**N (%)**	**N (%)**	**N (%)**	**N (%)**
**Subjects**	4430 (100)	7663 (100)	595 (100)	2137 (100)	3835 (100)	5526 (100)	2095 (100)
**Deprivation**							
**Affluent 1**	852 (19.2)	1501(19.6)	78 (13.1)	252 (11.8)	774 (20.2)	1249 (22.6)	435 (20.8)
**2**	913 (20.6)	1494 (19.5)	90 (15.1)	313 (14.7)	823 (21.5)	1181 (21.4)	452 (21.6)
**3**	852 (19.2)	1513 (19.7)	79 (13.3)	357 (16.7)	773 (20.2)	1156 (20.9)	438 (20.9)
**4**	891 (20.1)	1514 (19.8)	136 (22.9)	463 (21.7)	755 (19.7)	1051 (19.0)	387(18.5)
**Deprived 5**	879 (19.8)	1587 (20.7)	169 (28.4)	698 (32.7)	710 (18.5)	889 (16.1)	383(18.3)
**Not known**	43 (1.0)	54 (0.7)	43 (7.2)	54 (2.5)	-	-	-
	χ^2=6.00, p=0.31		χ^2=36.1, p<0.001		χ^2=15.1, p=0.004		
						χ^2=6.98, p=0.14	
					χ^2=10.9, p=0.028[b]		

[a] Controls were not selected for 595 cases (13.4%) whose parent(s) were not interviewed; and only one suitable control was identified for 7 of the remaining 3835 cases
[b] Participating cases (n=3835) *versus* all participating controls (n=7621)

For peer review only - http://bmjopen.bmj.com/site/about/guidelines.xhtml

Follow-up

The fact that basic details (matching variables, postcode, NHS number) of all cases and controls were registered and retained, regardless of the families' participation, enabled the conversion of the study into a matched cohort that could track all registered cases (n=4430) and their corresponding first-choice controls (n=7663) who were targeted for interview (**Table 1**; first two columns), as well as cases and controls whose parents participated and agreed to be interviewed (**Table 1**; last three columns).

Tracking all of registered cases and controls, the UKCCS now operates on a legal basis that permits follow-up information to be obtained from NHS administrative healthcare records without explicit consent, enabling all cases and their corresponding controls to be "flagged" at the national level and tracked via linkage to nationwide information on deaths, cancer registrations, and hospital episode data (inpatient day and overnight admissions; outpatient appointments and visits; accident and emergency presentations; and maternity admissions). Importantly, in line with current ethical and governance requirements, the matched-cohorts are now pseudonymized; NHS identifiers are no longer held by the study but by the national administrative bodies supplying linked data (NHS England, Public Health Scotland, NHS Central Register Scotland, and the Secured Anonymised Information Linkage Databank).

Beginning with the numbers registered in the original case-control study, the flow diagram presented in **Figure 1** gives the numbers of cases and controls who survived five years or more, followed by the numbers who were successfully linked to national administrative data sources**.** Cases enter the survivors' cohort exactly five years after their cancer was first diagnosed, and controls five years after their corresponding case's cancer was first diagnosed (pseudo-diagnosis date). Controls who emigrated, died or were lost to follow-up before five years had elapsed are not included in the survivor's cohort, nor are controls who were matched to cases who died within five years of their diagnosis; since, where applicable, their follow-up was truncated when their matched case died (**Figure 1**; bottom row). All those who were targeted for interview in the original case-control study are shown on the left-side of **Figure 1**, and all those whose parents were interviewed on the right-side; the total number of individuals that are being followed is shown in the yellow boxes in the centre of the figure.

Figure 1

With respect to the available data, the time periods currently covered by the various linked healthcare datasets, which are updated on an annual basis are shown in **Figure 2;** and the key data domains and fields are listed in **Box 1.**

Figure 2

For peer review only - http://bmjopen.bmj.com/site/about/guidelines.xhtml

Box 1: National administrative data available for comparative analysis: United Kingdom Childhood Cancer Study (UKCCS)

Source of data		Description of fields
Socio-demographic characteristics		Sex; year of birth; date of diagnosis/pseudo-diagnosis; indicators of deprivation at diagnosis/pseudo-diagnosis
Death notifications		Date and causes of death (ICD-10[a])
Emigrations		Date of embarkation
Cancer Registrations		Date of diagnosis; date of registration; topography (ICD revisions 7-10); morphology (ICD-O[b] revisions 1-3)
Secondary Care Data (Hospital Activity – HES[c]) Data		
	Inpatient and Day Cases	Date of admission; date of discharge; dates and types of procedures (OPCS-4[d], maximum 24); conditions at discharge (ICD-10[a], maximum 20); consultant specialties involved; source of referral; discharge destination
	Outpatient	Date of appointment and attendance flag; dates of procedures (maximum 24); consultant specialties involved
	Accident & Emergency	Date and reason for attendance; investigations and treatments; source of referral
	Maternity Inpatient and Day Cases	Date of admission to obstetrics or midwifery; date of discharge; conditions at discharge (ICD-10[a] maximum 20); age at birth; delivery method; babies' birthweights

[a]ICD: International Classification of Diseases.
[b]ICD-O: International Classification of Diseases for Oncology.
[c]HES: Hospital Episode Statistics in England, or equivalently, Scottish Morbidity Record (SMR) in Scotland, and the Patient Episode Database for Wales (PEDW).
[d]OPCS-4: Office of Population Censuses and Surveys Classification of Interventions and Procedures version 4.

Patient and public involvement and Engagement (PPIE)

The original case-control study was established over 30 years ago, at a time when lay-involvement in research was largely absent, and so did not benefit from formal links with patient/user groups. Members of the CCLG (Children's Cancer and Leukaemia Group, previously the UK Children's Cancer Study Group) were, however, fully involved throughout; and the study website (www.UKCCS.org) contains information about ongoing research activities and fair processing. The transformation of the study to focus on late effects among long-term childhood cancer survivors means that we can now move forwards to enable patients and the public to work in partnership with researchers and clinical staff to co-develop and deliver the research, disseminate findings, and determine the direction of future embedded studies. This input will ensure data are utilized in a way that secures maximize impact, improves long-term care, and promotes effective resource planning and management in the future.

Characteristics of individuals in the study

Detailed diagnostic and treatment information was obtained from multiple sources, including diagnosing/treatment centres, national treatment trials (where they existed), hospital records and reference laboratories. The diagnostic distribution, coded to the International Classification of Childhood Cancer 3rd edition (ICCC-3), as well as information on age, sex and interview status, is

For peer review only - http://bmjopen.bmj.com/site/about/guidelines.xhtml

shown in **Table 2**. As expected, given the increasing emphasis of leukaemia in later years, the final diagnostic distribution was weighted towards leukaemia. Variations with parental interview status are also evident; the non-interviewed group containing proportionally more CNS tumours (ICCC III) and other cancers (ICCC XI and XII). Among all registered cases, 1195 (27.0%) died within the first five years, and while in total, over 70% of the cases were alive at five years after diagnosis, the proportions within each ICCC-3 group varied with cancer (**Table 3**).

Table 2: Diagnostic frequencies, median ages, and parental interview status of children (0-14 years) registered in the United Kingdom Childhood Cancer Study (UKCCS)

Diagnosis (ICCC group)	Total N (%)	Males (%)	Age, median (interquartile range)	Interviewed	
				Yes N (%)	No N (%)
All cancers	4430(100.0)	55.7	5.1(2.7-9.7)	3835(100.0)	595(100.0)
Leukaemia (I)	1911(43.1)	55.8	4.5(2.8-8.0)	1734(45.2)	177(29.7)
Acute lymphoblastic leukaemia (Ia)	1580(35.7)	55.8	4.4(2.9-7.6)	1462(38.1)	118(19.8)
Acute myeloid leukaemia (Ib)	294(6.6)	53.7	5.4(1.8-11.2)	248(6.5)	46(7.7)
Lymphomas & reticuloendothelial (II)	413(9.3)	72.9	9.9(5.8-12.9)	357(9.3)	56(9.4)
Hodgkin lymphoma (IIa)	132(3.0)	71.2	11.6(7.9-13.5)	117(3.0)	15(2.5)
Non-Hodgkin lymphoma (IIb)	258(5.8)	72.9	9.4(5.2-12.0)	226(5.9)	32(5.4)
CNS, intracranial & intraspinal (III)	848(19.1)	50.1	6.5(3.3-10.1)	686(17.9)	162(27.2)
Ependymoma and choroid plexus (IIIa)	101(2.3)	58.4	3.4(1.4-9.0)	90(2.3)	11(1.8)
Astrocytoma (IIIb)	372(8.4)	44.1	6.9(4.0-10.5)	303(7.9)	69(11.6)
Intracranial & intraspinal embryonal (IIIc)	189(4.3)	55.6	6.0(2.7-8.8)	162(4.2)	27(4.5)
Neuroblastoma & other peripheral neural (IV)	222(5.0)	59.5	1.8(0.7-3.7)	188(4.9)	34(5.7)
Retinoblastoma (V)	112(2.5)	55.4	1.4(0.7-2.7)	87(2.3)	25(4.2)
Renal (VI)	221(5.0)	51.6	3.2(1.8-5.2)	199(5.2)	22(3.7)
Hepatic (VII)	36(0.8)	58.3	1.5(0.9-3.3)	32(0.8)	4(0.7)
Bone (VIII)	121(2.7)	53.7	11.2(8.9-13.5)	106(2.8)	15(2.5)
Soft tissue & other sarcomas (IX)	266(6.0)	54.9	5.7(2.8-10.6)	233(6.1)	33(5.5)
Germ cell & gonadal (X)	114(2.6)	54.4	6.9(1.4-12.3)	98(2.5)	16(2.7)
Other cancers (XI and XII)	99(2.2)	43.4	10.6(6.6-13.5)	62(1.6)	37(6.2)

For peer review only

For peer review only - http://bmjopen.bmj.com/site/about/guidelines.xhtml

Table 3: Numbers of children, numbers alive five years after diagnosis, and duration of follow-up (July 2020 England and Wales; June 2022 Scotland) by diagnosis (ICCC-3): UKCCS

Diagnosis (ICCC group)	Total	Alive 5-years after diagnosis[1]		Total Person-Years[2]	
		N	%	Median (IQR)	Total
**Total Cases**	4430	3212	72.5	25.3(3.5-27.1)	78,623
Leukaemias (I)	1911	1448	75.8	24.7(5.2-26.7)	34,423
Acute lymphoblastic leukaemia (Ia)	1580	1275	80.7	24.9(9.0-26.9)	30,206
Acute myeloid leukaemia (Ib)	294	161	54.8	19.4(0.9-25.8)	3,913
Lymphomas & reticuloendothelial (II)	413	344	83.3	26.1(20.4-27.5)	8,434
Hodgkin lymphoma (IIa)	132	127	96.2	27.3(26.1-28.2)	3,194
Non-Hodgkin lymphoma (IIb)	258	203	78.7	25.4(12.9-27.1)	4,880
CNS, intracranial and intraspinal (III)	848	534	63.0	23.3(1.3-26.9)	13,021
Ependymoma and choroid plexus (IIIa)	101	62	61.4	9.2(1.9-26.5)	1,359
Astrocytoma (IIIb)	372	275	73.9	26.2(3.6-27.5)	6,894
Intracranial embryonal (IIIc)	189	84	44.4	3.0(0.8-26.1)	2,050
Neuroblastoma and other peripheral neural (IV)	222	121	54.5	14.7(1.3-26.8)	3,109
Retinoblastoma (V)	112	108	96.4	26.8(26.0-27.5)	2,769
Renal (VI)	221	173	78.3	26.2(19.4-27.4)	4,477
Hepatic (VII)	36	27	75.0	25.8(2.6-26.9)	674
Bone (VIII)	121	70	57.9	20.0(1.9-26.9)	1,799
Soft tissue and other sarcomas (IX)	266	167	62.8	25.2(1.8-27.4)	4,205
Germ cell and gonadal (X)	114	92	80.7	26.5(18.7-27.5)	2,359
Other cancers (XI & XII)	99	70	70.7	26.5(2.4-27.7)	1,793
**Total Controls**	9758	9703	99.4	26.5(25.2-27.6)	244,061
All first choice	7663	7601	99.2	26.6(25.2-27.6)	191,484
Replacements (parents interviewed)	2095	2091	99.8	26.5(25.1-27.5)	52,577

[1]97.7% of cases and 97.1% of controls have been traced and have administrative data.
[2]Person-years are calculated for the total number of cases from diagnosis to end of follow-up, and for controls from their pseudo-diagnosis date, i.e. the date they were the same age as their matched case was at diagnosis

FINDINGS TO DATE

The UKCCS provides a valuable resource within which to examine both the potential causes and long-term health consequences of childhood cancer. Thus far, over 90 peer-reviewed articles containing UKCCS data have been published, and a full list is on the study website (www.UKCCS.org). Assembling data from multiple sources, key reports include ALL genome-wide association studies (GWAS)[14,15], and those examining the potential aetiological roles of a wide range of biological (e.g. infections/markers of infectious exposure)[16–18], physical (e.g. ionizing/non-ionizing radiation)[19–22] and chemical (e.g. prescription drugs/smoking) agents acting on parents before the child's conception and the child during pregnancy/early life[13,23,24], as well as relationships with other illnesses (e.g. allergy) and birth characteristics (e.g. birthweight)[16,25,26]. Importantly, as noted above, the UKCCS accessed medical records to help answer key questions relating to prescription drugs, illness histories and co-morbidities; such contemporaneously recorded information, which by its nature is free from differential recall bias, often producing very different results from that obtained by self-report[17,27–29].

With respect to the longer-term health and healthcare needs of childhood cancer survivors, the conversion of the UKCCS into a matched cohort is now yielding important results about the

For peer review only - http://bmjopen.bmj.com/site/about/guidelines.xhtml

morbidity and mortality of childhood cancer survivors. Early findings based on the case-cohort alone highlighted survival variations with age and sex across a range of subtypes[9], and for acute lymphoblastic leukaemia, survival associations with area-based measures of deprivation at the time of diagnosis were also noted[10]. More recently, we have begun examining secondary care hospital activity patterns, comparing findings in the case-cohort to those in the comparison cohort over the first 25 years. Adding to excess risks of death and cancer, our first report demonstrated that survivors of childhood acute lymphoblastic leukaemia (ALL) experienced excess outpatient and inpatient activity across their teenage and young adult years; which was not related to routine follow-up monitoring. Indeed, with hospital activity being higher than expected in specialties covering most organ and tissue systems, survivors were more than twice as likely to fall under the care of endocrinology, cardiology, respiratory medicine, ophthalmology, neurology and/or gastroenterology specialists[11]. Furthermore, these differences showed no signs of diminishing in the first 25 years of follow-up, underscoring the need to take prior cancer drug and/or radiation treatment into account when interpreting seemingly unrelated symptoms in later life.

STRENGTHS AND LIMITATIONS

Covering England, Scotland and Wales, findings from this population-based age- and sex- matched cohort study are based on routinely compiled linked health data, not self-report. With minimal selection bias and loss to follow-up,the matched-cohort design allows more granular analyses to be performed than is possible in case-cohorts that rely solely on general population rates, as has been done in other UK-based studies[30–33]. For example, at the point of cohort entry the individually age- and sex-matched comparison cohort has a similar deprivation distribution to the case-cohort, providing a robust baseline against which to evaluate the potential impact of deprivation across the life-course. In addition, because the UKCCS cohorts can be divided into those whose families participated in the original case-control study and those who did not, the study is well-placed to investigate the impact of selection bias; an analysis which most cohorts cannot undertake, largely because they were not designed to do so, most being either record-based (as on the left side of **Figure 1**) [34–37]**,** or interview-based (as on the right-side of **Figure 1**) [38,39]. Thus far, with follow-up extending to over 25 years since diagnosis, we have examined health from childhood through TYA and early adulthood, and both cohorts will continue to be tracked as they age. With respect to limitations, lack of primary care data and information from psychosocial services are obvious deficiencies that currently affects all UK record-linkage studies of the type described here [40–42]. Analyses are also constrained by the fact that national administrative healthcare data are primarily collected for administrative and clinical purposes, and not for research.

DATA SHARING

Reasonable requests for data sharing will be considered by the authors.

Ethics approval

The United Kingdom Childhood Cancer Study (UKCCS) has ethics approval from the Yorkshire & the Humber-Leeds West Research Ethics Committee (reference 18/YH/0135) and exemption from Section 251 of the Health & Social Care Act (reference 18/CAG/0066).

Author contributions

ER contributed to the design of the original case-control study, initiated the matched cohort, and is the guarantor. SK and JS have also been involved with the study since its inception. EK was involved in initiating the matched cohort and leads on cohort data management. AB, EK, JS, and AS have conducted many of the analyses. SK is the clinical lead, and DH and RS have extensive experience of working with patients and carers to deliver on PPIE. All authors have contributed to this report.

For peer review only - http://bmjopen.bmj.com/site/about/guidelines.xhtml

For peer review only

Funding
The UKCCS work reported on here was previously supported by Blood Cancer UK grant number 15037, and is jointly now supported by Blood Cancer UK and Cancer Research UK (grant number 29685). AB is supported by a fellowship from the Fondation ARC pour la Recherche sur le Cancer (PDF20190508759). The funders had no role in considering the study design or in the collection, analysis, interpretation of data, writing of the manuscript, or the decision to submit the article for publication.

Conflicts of interest
None of the authors have any conflicts of interest

References

1 Gatta G, Botta L, Rossi S, *et al.* Childhood cancer survival in Europe 1999-2007: results of EUROCARE-5--a population-based study. *Lancet Oncol* 2014;**15**:35–47. doi:10.1016/S1470-2045(13)70548-5

2 American Cancer Society. Cancer Facts & Figures 2015. Atlanta, GA, USA: : American Cancer Society, Inc. 2015. https://www.cancer.org/research/cancer-facts-statistics/all-cancer-facts-figures/cancer-facts-figures-2015.html (accessed 2 Dec 2022).

3 The European Society for Paediatric Oncology. SIOP Europe Strategic Plan. Update (2021-2026). A European Cancer Plan for Children and Adolescents. Brussels, Belgium: : SIOP Europe 2021. https://siope.eu/european-strategic-plan/

4 Kebudi R, Özdemir GN. Second malignant neoplasms in childhood cancer survivors. *Curr Pediatr Rev* 2017;**13**:34–41.

5 Fidler MM, Reulen RC, Henson KE, *et al.* Population-Based Long-Term Cardiac-Specific Mortality Among 34,489 Five-Year Survivors of Childhood Cancer in Great Britain. *Circulation* 2017;**135**:951–63. doi:10.1161/CIRCULATIONAHA.116.024811

6 Dietz AC, Chen Y, Yasui Y, *et al.* Risk and impact of pulmonary complications in survivors of childhood cancer: A report from the Childhood Cancer Survivor Study. *Cancer* 2016;**122**:3687–96. doi:10.1002/cncr.30200

7 Overbeek A, van den Berg MH, van Leeuwen FE, *et al.* Chemotherapy-related late adverse effects on ovarian function in female survivors of childhood and young adult cancer: A systematic review. *Cancer Treat Rev* 2017;**53**:10–24. doi:10.1016/j.ctrv.2016.11.006

8 UK Childhood Cancer Study Investigators. The United Kingdom Childhood Cancer Study: objectives, materials and methods. *Br J Cancer* 2000;**82**:1073–102. doi:10.1054/bjoc.1999.1045

9 Johnston WT, Lightfoot TJ, Simpson J, *et al.* Childhood cancer survival: a report from the United Kingdom Childhood Cancer Study. *Cancer Epidemiol* 2010;**34**:659–66. doi:10.1016/j.canep.2010.06.020

10 Lightfoot TJ, Johnston WT, Simpson J, *et al.* Survival from childhood acute lymphoblastic leukaemia: the impact of social inequality in the United Kingdom. *Eur J Cancer* 2012;**48**:263–9. doi:10.1016/j.ejca.2011.10.007

For peer review only - http://bmjopen.bmj.com/site/about/guidelines.xhtml

11 Kane E, Kinsey S, Bonaventure A, *et al.* Excess morbidity and mortality among survivors of childhood acute lymphoblastic leukaemia: 25 years of follow-up from the United Kingdom Childhood Cancer Study (UKCCS) population-based matched cohort. *BMJ Open* 2022;**12**:e056216. doi:10.1136/bmjopen-2021-056216

12 Smith A, Roman E, Simpson J, *et al.* Childhood leukaemia and socioeconomic status: fact or artefact? A report from the United Kingdom childhood cancer study (UKCCS). *International Journal of Epidemiology* 2006;**35**:1504–13. doi:10.1093/ije/dyl193

13 Roman E, Simpson J, Ansell P, *et al.* Perinatal and reproductive factors: a report on haematological malignancies from the UKCCS. *Eur J Cancer* 2005;**41**:749–59. doi:10.1016/j.ejca.2004.11.006

14 Papaemmanuil E, Hosking FJ, Vijayakrishnan J, *et al.* Loci on 7p12.2, 10q21.2 and 14q11.2 are associated with risk of childhood acute lymphoblastic leukemia. *Nat Genet* 2009;**41**:1006–10. doi:10.1038/ng.430

15 Vijayakrishnan J, Kumar R, Henrion MYR, *et al.* A genome-wide association study identifies risk loci for childhood acute lymphoblastic leukemia at 10q26.13 and 12q23.1. *Leukemia* 2017;**31**:573–9. doi:10.1038/leu.2016.271

16 Roman E, Simpson J, Ansell P, *et al.* Childhood Acute Lymphoblastic Leukemia and Infections in the First Year of Life: A Report from the United Kingdom Childhood Cancer Study. *American Journal of Epidemiology* 2007;**165**:496–504. doi:10.1093/aje/kwk039

17 Simpson J, Smith A, Ansell P, *et al.* Childhood leukaemia and infectious exposure: A report from the United Kingdom Childhood Cancer Study (UKCCS). *European Journal of Cancer* 2007;**43**:2396–403. doi:10.1016/j.ejca.2007.07.027

18 Crouch S, Lightfoot T, Simpson J, *et al.* Infectious illness in children subsequently diagnosed with acute lymphoblastic leukemia: modeling the trends from birth to diagnosis. *American journal of epidemiology* 2012;**176**:402–8.

19 Ahlbom A, Day N, Feychting M, *et al.* A pooled analysis of magnetic fields and childhood leukaemia. *Br J Cancer* 2000;**83**:692–8. doi:10.1054/bjoc.2000.1376

20 Skinner J, Mee TJ, Blackwell RP, *et al.* Exposure to power frequency electric fields and the risk of childhood cancer in the UK. *Br J Cancer* 2002;**87**:1257–66. doi:10.1038/sj.bjc.6600602

21 UK Childhood Cancer Study Investigators. The United Kingdom Childhood Cancer Study of exposure to domestic sources of ionising radiation: 1: radon gas. *Br J Cancer* 2002;**86**:1721–6. doi:10.1038/sj.bjc.6600276

22 UK Childhood Cancer Study Investigators. The United Kingdom Childhood Cancer Study of exposure to domestic sources of ionising radiation: 2: gamma radiation. *Br J Cancer* 2002;**86**:1727–31. doi:10.1038/sj.bjc.6600277

23 Roman E, Fear NT, Ansell P, *et al.* Vitamin K and childhood cancer: analysis of individual patient data from six case–control studies. *British Journal of Cancer* 2002;**86**:63–9.

24 Bonaventure A, Simpson J, Ansell P, *et al.* Prescription drug use during pregnancy and risk of childhood cancer – Is there an association? *Cancer Epidemiology* 2015;**39**:73–8. doi:10.1016/j.canep.2014.10.008

For peer review only

25 Bonaventure A, Simpson J, Ansell P, *et al.* Paediatric acute lymphoblastic leukaemia and caesarean section: A report from the United Kingdom Childhood Cancer Study (UKCCS). *Paediatric and Perinatal Epidemiology* 2020;**34**:344–9. doi:10.1111/ppe.12662

26 Milne E, Greenop KR, Metayer C, *et al.* Fetal growth and childhood acute lymphoblastic leukemia: Findings from the childhood leukemia international consortium. *International Journal of Cancer* 2013;**133**:2968–79. doi:10.1002/ijc.28314

27 Roman E, Simpson J, Ansell P, *et al.* Infectious proxies and childhood leukaemia: Findings from the United Kingdom Childhood Cancer Study (UKCCS). *Blood Cells, Molecules, and Diseases* 2009;**42**:126–8. doi:10.1016/j.bcmd.2008.10.007

28 Smith A, Roman E, Simpson J. Childhood leukaemia and socioeconomic status. *International Journal of Epidemiology* 2007;**36**:1156. doi:10.1093/ije/dym166

29 Bonaventure A, Kane E, Simpson J, *et al.* Maternal infections and medications in pregnancy: how does self-report compare to medical records in childhood cancer case–control studies? *International Journal of Epidemiology* 2023;:dyad019. doi:10.1093/ije/dyad019

30 van Laar M, Feltbower RG, Gale CP, *et al.* Cardiovascular sequelae in long-term survivors of young peoples' cancer: a linked cohort study. *Br J Cancer* 2014;**110**:1338–41. doi:10.1038/bjc.2014.37

31 Reulen RC, Bright CJ, Winter DL, *et al.* Pregnancy and Labor Complications in Female Survivors of Childhood Cancer: The British Childhood Cancer Survivor Study. *J Natl Cancer Inst* 2017;**109**. doi:10.1093/jnci/djx056

32 Smith L, Glaser AW, Peckham D, *et al.* Respiratory morbidity in young people surviving cancer: Population-based study of hospital admissions, treatment-related risk factors and subsequent mortality. *Int J Cancer* 2019;**145**:20–8. doi:10.1002/ijc.32066

33 Reulen RC, Guha J, Bright CJ, *et al.* Risk of cerebrovascular disease among 13 457 five-year survivors of childhood cancer: A population-based cohort study. *International Journal of Cancer* 2021;**148**:572–83. doi:10.1002/ijc.33218

34 McBride ML, Rogers PC, Sheps SB, *et al.* Childhood, adolescent, and young adult cancer survivors research program of British Columbia: objectives, study design, and cohort characteristics. *Pediatr Blood Cancer* 2010;**55**:324–30. doi:10.1002/pbc.22476

35 Asdahl PH, Winther JF, Bonnesen TG, *et al.* The Adult Life After Childhood Cancer in Scandinavia (ALiCCS) Study: Design and Characteristics. *Pediatr Blood Cancer* 2015;**62**:2204–10. doi:10.1002/pbc.25661

36 Kirchhoff AC, Fluchel MN, Wright J, *et al.* Risk of Hospitalization for Survivors of Childhood and Adolescent Cancer. *Cancer Epidemiol Biomarkers Prev* Published Online First: 12 June 2014. doi:10.1158/1055-9965.EPI-13-1090

37 Streefkerk N, Tissing WJE, Korevaar JC, *et al.* A detailed insight in the high risks of hospitalizations in long-term childhood cancer survivors-A Dutch LATER linkage study. *PLoS ONE* 2020;**15**:e0232708. doi:10.1371/journal.pone.0232708

For peer review only - http://bmjopen.bmj.com/site/about/guidelines.xhtml

38 Robison LL, Mertens AC, Boice JD, *et al.* Study design and cohort characteristics of the Childhood Cancer Survivor Study: a multi-institutional collaborative project. *Med Pediatr Oncol* 2002;**38**:229–39. doi:10.1002/mpo.1316

39 Hawkins MM, Lancashire ER, Winter DL, *et al.* The British Childhood Cancer Survivor Study: Objectives, methods, population structure, response rates and initial descriptive information. *Pediatr Blood Cancer* 2008;**50**:1018–25. doi:10.1002/pbc.21335

40 Chow EJ, Ness KK, Armstrong GT, *et al.* Current and coming challenges in the management of the survivorship population. *Semin Oncol* 2020;**47**:23–39. doi:10.1053/j.seminoncol.2020.02.007

41 Andrés-Jensen L, Skipper MT, Mielke Christensen K, *et al.* National, clinical cohort study of late effects among survivors of acute lymphoblastic leukaemia: the ALL-STAR study protocol. *BMJ Open* 2021;**11**. doi:10.1136/bmjopen-2020-045543

42 van Erp LME, Maurice-Stam H, Kremer LCM, *et al.* A vulnerable age group: the impact of cancer on the psychosocial well-being of young adult childhood cancer survivors. *Support Care Cancer* 2021;**29**:4751–61. doi:10.1007/s00520-021-06009-y

For peer review only

For peer review only - http://bmjopen.bmj.com/site/about/guidelines.xhtml

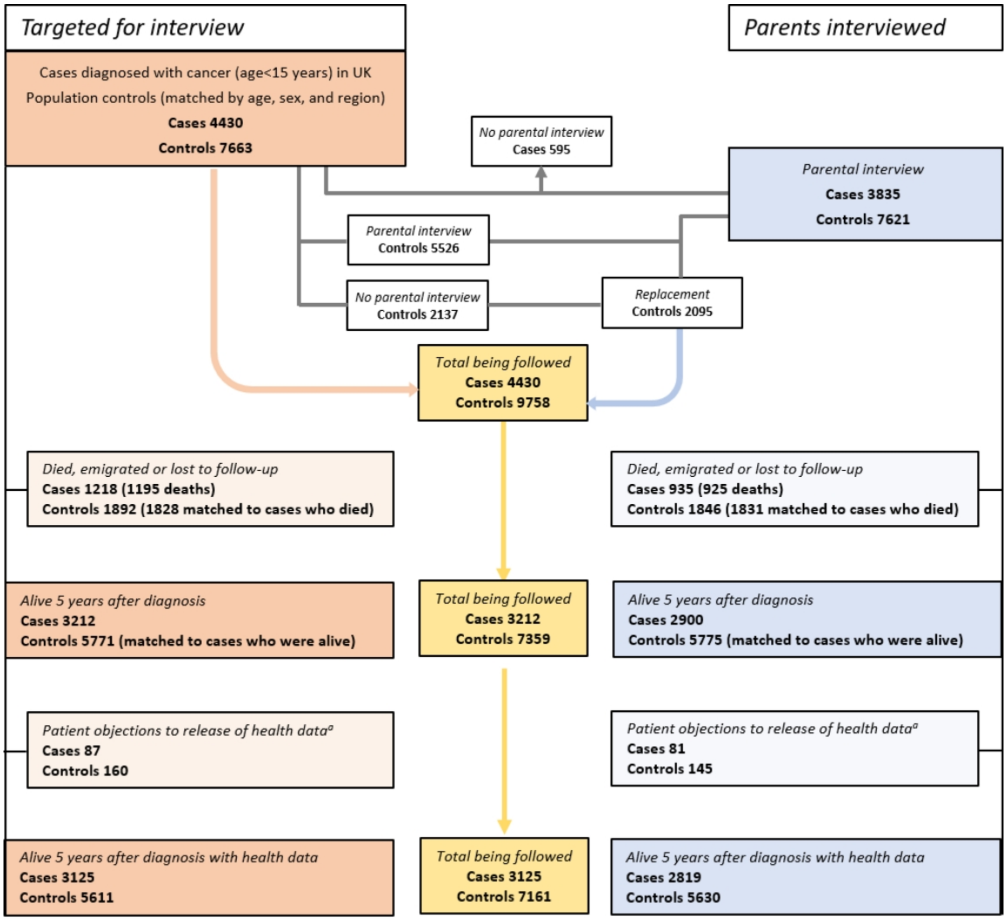

[a]A patient objection (also known as a Type 2 objection) is a request expressed by a registered patient lodged with a GP Practice that indicates that personal identifiable information that relates to the patient must not be disseminated or published, and as such the UKCCS does not receive their health data. This is only applicable in England and Wales.

Figure 1 Flowchart showing UKCCS subjects distributed by parental interview status (total targeted for interview and total interviewed) and follow-up status; the numbers of 5-year survivors with linked health data are presented in the bottom row

168x167mm (300 x 300 DPI)

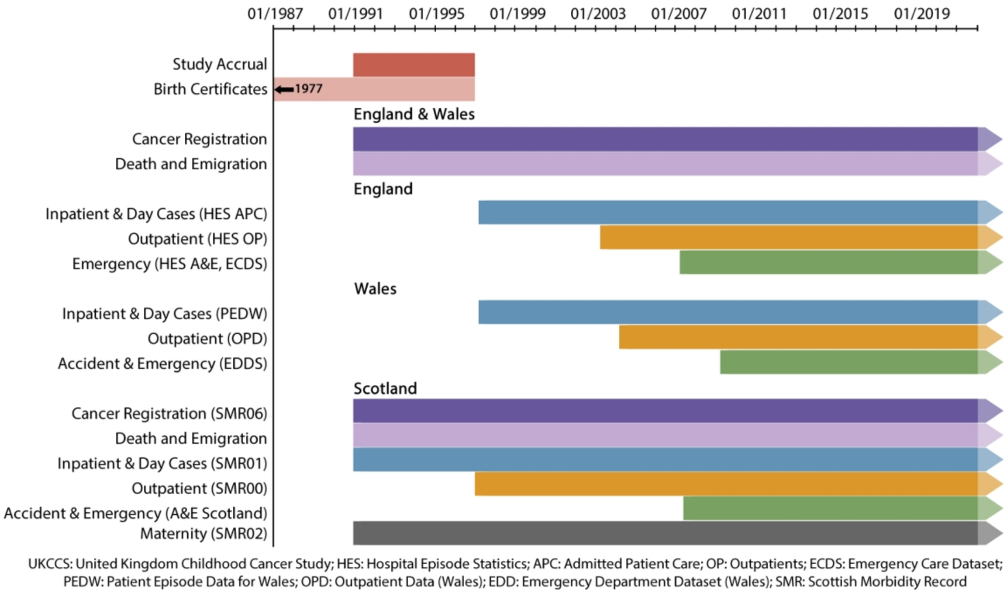

Figure 2 Linkage to national administrative datasets: United Kingdom Childhood Cancer Study (UKCCS)

287x167mm (300 x 300 DPI)

STROBE 2007 (v4) Statement—Checklist of items that should be included in reports of *cohort studies*

Section/Topic	Item #	Recommendation	Reported on page #
**Title and abstract**	1	(*a*) Indicate the study's design with a commonly used term in the title or the abstract	1-2
		(*b*) Provide in the abstract an informative and balanced summary of what was done and what was found	2
**Introduction**			
Background/rationale	2	Explain the scientific background and rationale for the investigation being reported	2-4
Objectives	3	State specific objectives, including any prespecified hypotheses	2
**Methods**			
Study design	4	Present key elements of study design early in the paper	2
Setting	5	Describe the setting, locations, and relevant dates, including periods of recruitment, exposure, follow-up, and data collection	2-5
Participants	6	(*a*) Give the eligibility criteria, and the sources and methods of selection of participants. Describe methods of follow-up	3-4
		(*b*) For matched studies, give matching criteria and number of exposed and unexposed	3
Variables	7	Clearly define all outcomes, exposures, predictors, potential confounders, and effect modifiers. Give diagnostic criteria, if applicable	3-6
Data sources/ measurement	8*	For each variable of interest, give sources of data and details of methods of assessment (measurement). Describe comparability of assessment methods if there is more than one group	3-6
Bias	9	Describe any efforts to address potential sources of bias	3-6
Study size	10	Explain how the study size was arrived at	3-8
Quantitative variables	11	Explain how quantitative variables were handled in the analyses. If applicable, describe which groupings were chosen and why	3-7
Statistical methods	12	(*a*) Describe all statistical methods, including those used to control for confounding	3-6
		(*b*) Describe any methods used to examine subgroups and interactions	5
		(*c*) Explain how missing data were addressed	5
		(*d*) If applicable, explain how loss to follow-up was addressed	5
		(*e*) Describe any sensitivity analyses	NA
**Results**			

For peer review only

Participants	13*	(a) Report numbers of individuals at each stage of study—eg numbers potentially eligible, examined for eligibility, confirmed eligible, included in the study, completing follow-up, and analysed	5
		(b) Give reasons for non-participation at each stage	3-5
		(c) Consider use of a flow diagram	3
Descriptive data	14*	(a) Give characteristics of study participants (eg demographic, clinical, social) and information on exposures and potential confounders	4,7,8
		(b) Indicate number of participants with missing data for each variable of interest	8
		(c) Summarise follow-up time (eg, average and total amount)	8
Outcome data	15*	Report numbers of outcome events or summary measures over time	8
Main results	16	(*a*) Give unadjusted estimates and, if applicable, confounder-adjusted estimates and their precision (eg, 95% confidence interval). Make clear which confounders were adjusted for and why they were included	NA
		(*b*) Report category boundaries when continuous variables were categorized	NA
		(*c*) If relevant, consider translating estimates of relative risk into absolute risk for a meaningful time period	NA
Other analyses	17	Report other analyses done—eg analyses of subgroups and interactions, and sensitivity analyses	NA
**Discussion**			
Key results	18	Summarise key results with reference to study objectives	8-9
**Limitations**			
Interpretation	20	Give a cautious overall interpretation of results considering objectives, limitations, multiplicity of analyses, results from similar studies, and other relevant evidence	8-9
Generalisability	21	Discuss the generalisability (external validity) of the study results	9
**Other information**			
Funding	22	Give the source of funding and the role of the funders for the present study and, if applicable, for the original study on which the present article is based	10

*Give information separately for cases and controls in case-control studies and, if applicable, for exposed and unexposed groups in cohort and cross-sectional studies.

Note: An Explanation and Elaboration article discusses each checklist item and gives methodological background and published examples of transparent reporting. The STROBE checklist is best used in conjunction with this article (freely available on the Web sites of PLoS Medicine at http://www.plosmedicine.org/, Annals of Internal Medicine at http://www.annals.org/, and Epidemiology at http://www.epidem.com/). Information on the STROBE Initiative is available at www.strobe-statement.org.

For peer review only

BMJ Open

Cohort Profile: The United Kingdom Childhood Cancer Study (UKCCS): a UK-wide population-based study examining the health of cancer survivors

Journal:	*BMJ Open*
Manuscript ID	bmjopen-2023-073712.R1
Article Type:	Cohort profile
Date Submitted by the Author:	18-Aug-2023
Complete List of Authors:	Roman, Eve; University of York, Health Sciences<br>Kane, Eleanor; University of York, Health Sciences<br>Smith, Alexandra; University of York, Health Sciences<br>Howell, Debra; University of York, Health Sciences<br>Sheridan, Rebecca; University of York,<br>Simpson, Jill; University of York<br>Bonaventure, Audrey; INSERM<br>Kinsey, Sally; Leeds Children's Hospital, Paediatric Haematology
**Primary Subject Heading**:	Public health
Secondary Subject Heading:	Epidemiology
Keywords:	Leukaemia < HAEMATOLOGY, Lymphoma < ONCOLOGY, EPIDEMIOLOGY, PUBLIC HEALTH, Quality of Life, Risk Factors

SCHOLARONE™ Manuscripts

I, the Submitting Author has the right to grant and does grant on behalf of all authors of the Work (as defined in the below author licence), an exclusive licence and/or a non-exclusive licence for contributions from authors who are: i) UK Crown employees; ii) where BMJ has agreed a CC-BY licence shall apply, and/or iii) in accordance with the terms applicable for US Federal Government officers or employees acting as part of their official duties; on a worldwide, perpetual, irrevocable, royalty-free basis to BMJ Publishing Group Ltd ("BMJ") its licensees and where the relevant Journal is co-owned by BMJ to the co-owners of the Journal, to publish the Work in this journal and any other BMJ products and to exploit all rights, as set out in our licence.

The Submitting Author accepts and understands that any supply made under these terms is made by BMJ to the Submitting Author unless you are acting as an employee on behalf of your employer or a postgraduate student of an affiliated institution which is paying any applicable article publishing charge ("APC") for Open Access articles. Where the Submitting Author wishes to make the Work available on an Open Access basis (and intends to pay the relevant APC), the terms of reuse of such Open Access shall be governed by a Creative Commons licence – details of these licences and which Creative Commons licence will apply to this Work are set out in our licence referred to above.

Other than as permitted in any relevant BMJ Author's Self Archiving Policies, I confirm this Work has not been accepted for publication elsewhere, is not being considered for publication elsewhere and does not duplicate material already published. I confirm all authors consent to publication of this Work and authorise the granting of this licence.

For peer review only

Cohort Profile: The United Kingdom Childhood Cancer Study (UKCCS): a UK-wide population-based study examining the health of cancer survivors

Eve Roman[1*]

Eleanor Kane[1]

Alexandra Smith[1]

Debra Howell[1]

Rebecca Sheridan [1]

Jill Simpson[1]

Audrey Bonaventure[2]

Sally Kinsey[1]

[1] Epidemiology and Cancer Statistics Group, Department of Health Sciences, University of York, YO10 5DD, UK

*Corresponding author: eve.roman@york.ac.uk

Word count: Main text = 3766

For peer review only - http://bmjopen.bmj.com/site/about/guidelines.xhtml

Abstract

Purpose: The United Kingdom Childhood Cancer Study's (UKCCS's) matched-cohort was established to examine the longer-term morbidity and mortality of individuals previously diagnosed with cancer before 15 years of age; comparing future healthcare patterns in 5-year cancer survivors to baseline activity seen in age- and sex-matched individuals from the general population.

Participants: Predicated on a national childhood cancer case-control study conducted in the early 1990s (4430 cases, 9758 controls) in England, Scotland and Wales, the case-population comprises 3125 cancer survivors (>5 years), and the control-population 7161 age- and sex-matched individuals from the general population who did not have cancer as a child. Participants are now being followed-up via linkage to national administrative healthcare databases (deaths, cancers, and secondary-care hospital activity).

Findings to date: Enabling the creation of cohorts with minimal selection bias and loss to follow-up, the original case-control study registered all newly diagnosed cases of childhood cancer and their corresponding controls, regardless of their family's participation. Early findings based on the registered case-population found marked survival variations with age and sex across subtypes, and differences with deprivation among acute lymphoblastic leukaemia (ALL) survivors. More recently, comparing the health-activity patterns of the case- and control-populations, revealed that survivors of childhood ALL experienced excess outpatient and inpatient activity across their teenage/young adult years. Adding to increased risks of cancer and death and involving most clinical specialties, excesses were not related to routine follow-up monitoring, and showed no signs of diminishing over time.

Future plans: With annual linkage updates, the UKCCS's maturing population-based matched-cohorts provide the foundation for tracking the health of individuals through their lifetime. Comparing the experience of childhood cancer survivors to that of unaffected general-population counterparts, this will include examining subsequent morbidity and mortality, secondary care hospital activity, and the impact of deprivation on longer-term outcomes.

Strengths and limitations of this study (max 5 bullet points)

- Covering England, Scotland and Wales, findings from this national study are based on routinely compiled linked health data, not self-report.
- Regardless of whether or not their families participated in the original case-control study, all children are included in the matched cohort, enabling the investigation of selection bias.
- With minimal selection bias and loss to follow-up, the individually age- and sex-matched control-population has a similar deprivation distribution to the case-population, providing a robust baseline against which to evaluate the impact of deprivation across the life-course.
- Analyses are constrained by the fact that national administrative healthcare data are primarily collected for administrative and clinical purposes, and not for research.
- With respect to weaknesses, lack of primary care data and information from psychosocial services are obvious deficiencies that currently affects all UK record-linkage studies of the type described here.

For peer review only - http://bmjopen.bmj.com/site/about/guidelines.xhtml

INTRODUCTION

In recent decades, advances in molecular biology and therapy have transformed the landscape for several childhood cancers, changing many from rapidly fatal diseases to treatable conditions with a good prognosis; overall survival now exceeds 80% in the UK and other economically developed countries[1,2]. Accordingly, it is estimated that by 2030 there will be around three-quarters of a million childhood cancer survivors in Europe alone; this number increasing as therapies improve and populations age[3]. Whilst the majority of cancers in children are more responsive to chemotherapy than those in adults, treatments are often more aggressive, and adverse health problems are well-known to occur later in life (e.g. second cancers, cardiac and bone problems, and fertility issues)[4–7].

With a view to investigating childhood cancer survivors' health, the national matched-cohort described in this report was established with the aim of examining the relationship between cancers diagnosed before 15 years of age and other morbidities and health-states in the years after cancer diagnosis; specific aims including the examination of secondary care hospital activity patterns, and the impact of deprivation on longer-term outcomes. The cohorts are predicated on the structures established in the United Kingdom Childhood Cancer Study (UKCCS), which was set-up as a national population-based case-control study in the early 1990s in England, Scotland and Wales to investigate a wide range of possible causes of childhood cancer. Collecting data from multiple sources (including interviews with parents, primary care records, obstetric/neonatal notes, birth certificates, household radiation measurements, and pre-treatment and remission blood samples), the case-control study investigated the potentially carcinogenic effects of a wide-range of physical (e.g. non-ionizing radiation), chemical (e.g. drugs) and biological (e.g. infectious agents) agents [8–14]. Examining associations in the pre-natal, in-utero, and post-natal periods, as well as associations with birth characteristics (e.g. birthweight) and other illnesses (e.g. allergies), over 90 reports have thus far been published (www.UKCCS.org).

Underpinned by updated ethics and legal permissions, the UKCCS has now been transformed into a matched population-based cohort, with follow-up linkages to electronic national administrative healthcare databases (inpatient and outpatient hospital episode statistics – HES, cancers, and deaths) [15–17]. Importantly, all cases and controls (regardless of parents' participation in the original case-control study) are being tracked forwards in time via annually updated linkages; the comparison cohort (controls) enabling robust baseline effect measures to be estimated. Currently over 25 years of follow-up data are available with which to investigate health-care patterns and health events occurring in cancer survivors. The children in the original UKCCS are presently aged between 27-46 years, and this report describes the study's underpinning cohort methods, and summarises some of the initial findings.

COHORT DESCRIPTION

Full details of the UKCCS case-control study (ukccs.org) which provides the foundation of the matched-cohort described here have been published[8,10,18]. Briefly, the sampling frame for cases and controls comprised all children registered with the National Health Service (NHS) in England, Scotland or Wales at the time the study was conducted (>98% of the childhood population in these countries); unfortunately for logistical reasons Northern Ireland (~3.5% of the total UK 0-14 population) was not included.

Overseen by a management committee that included epidemiologists, statisticians, paediatric oncologist/haematologists and expert scientists that was chaired by Professor Sir Richard Doll, 10

For peer review only - http://bmjopen.bmj.com/site/about/guidelines.xhtml

UKCCS administrative areas covering the whole of Britain were created for the purposes of data collection, with each being the responsibility of a well-established epidemiological research centre[8] (**Supplementary Figure 1**).

The study began on 1 January 1991 in Scotland, and either 1 April 1992 or 1 September 1992 in the nine UKCCS administrative areas of England and Wales. In Scotland, case accrual ended on 31 December 1994, and in England and Wales, it was restricted to children diagnosed with leukaemia or non-Hodgkin lymphoma throughout 1995, and leukaemia alone throughout 1996. In order to ensure ascertainment completeness, proactive cancer notification systems were established in all diagnostic/treatment centres across the UK; the majority of cases being notified at the point of diagnosis directly to the relevant UKCCS administrative centre by paediatric oncologists or haematologists practising in the study area. Subsequent crosschecks were made against regional childhood cancer registries (where they existed); the population-based National Registry of Childhood Tumours (NRCT), which covered England, Scotland and Wales [8,19]; and clinical trial datasets (acute lymphoblastic leukaemia).

With the permission of the child's clinical team, the parents of children with cancer were subsequently contacted. Each child whose parents agreed to be interviewed (3835/4430; 87%) was individually matched on sex, date of birth (month and year) and UKCCS region of residence to 10 controls that were randomly selected from the same population-based health service authority list as the case. With their general practitioner's (GP's) permission, the parents of the first two controls on the list ("first-choice" controls) were contacted and asked to participate in the study; but if the GP declined, or the parents did not wish to be interviewed, the next child was selected, and this procedure was repeated until two control families agreed to take part. By the end of the data collection phase, 96.7% (3786/3835) cases had two participating controls and 1.3% (49/3835) had one.

In order to monitor the characteristics of respondents and non-respondents across the study, and examine the potential impact of selection/participation bias, the registration details of all cases and controls were retained, regardless of whether or not their families were interviewed. Using standard methods, address postcodes were used to allocate deprivation scores for the home at diagnosis (all registered cases) or pseudo-diagnosis (all registered controls; date coinciding with the exact age that the corresponding case was diagnosed); and also at the time they were born (using mother's address on the child's birth certificate). Full details of the methods used have been previously published [8,18]. Briefly, deprivation categories were then derived by dividing the continuous score for the national 1991 census enumeration areas into five equally-sized groups, with group one representing the most affluent and group five the most deprived.

Illustrating the UKCCS's potential to examine issues relating to selection bias, the first two columns of **Table 1** distribute all registered subjects in the case-control study according to the area-based deprivation score of their home at diagnosis (cases) or pseudo-diagnosis (controls; date coinciding with the exact age that the corresponding case was diagnosed). With roughly 20% of the population in each quintile, the deprivation distribution of the 5-year survivor cohorts (last two columns of **Table 1,** right-side) mirror those of the case-control study (**Table 1,** left-side).

For peer review only - http://bmjopen.bmj.com/site/about/guidelines.xhtml

Table 1: Deprivation at cancer diagnosis/pseudo-diagnosis of subjects identified for inclusion on the case-control study and those for entry into the matched-cohorts

	Case-control-study: Subjects targeted for inclusion		Five-year Survivor cohorts	
	**Cases**	**First-choice controls**[1]	**Cases**	**First choice controls**
	N (%)	N (%)	N (%)	N (%)
**Deprivation**	4430 (100)	7663 (100)	3125 (100)	5611 (100)
**Affluent 1**	852 (19.2)	1501(19.6)	613(19.6)	1094(19.5)
**2**	913 (20.6)	1494 (19.5)	666(21.3)	1113(19.8)
**3**	852 (19.2)	1513 (19.7)	620(19.8)	1080(19.2)
**4**	891 (20.1)	1514 (19.8)	604(19.3)	1141(20.3)
**Deprived 5**	879 (19.8)	1587 (20.7)	604(19.3)	1140(20.3)
**Not known**	43 (1.0)	54 (0.7)	18(0.6)	43(0.8)
	χ^2=6.00, p=0.31		χ^2=5.57, p=0.35	

[1]Controls were not selected for 595 cases (13.4%) whose parent(s) were not interviewed; and only one suitable first-choice control was identified for 7 of the remaining 3835 cases.

As expected, the deprivation distribution of those whose parents participated in the case-control study differed from those who did not (**Table 2**). With 32.7% of non-participating controls residing in the most deprived quintile, the effect was more pronounced than in the case population (28.4%). Exacerbated by the fact that participation among case families was higher (3835/4430=86.6%) than among first-choice control families (5526/7663=72.1%), the deprivation distributions of interviewed cases (n=3835, column 1) and first-choice controls (n= 5526, column 3) differ significantly from each other (χ^2=15.1, p=0.004). As expected, distributing individuals in the 5-year survivors cohort by the same categories produced similar differences (**Table 2,** right-side). Such categorization, will not only allow us to evaluate the potential impact of deprivation at birth and diagnosis on hospital activity among 5-year survivors, but also to examine selection bias.

For peer review only - http://bmjopen.bmj.com/site/about/guidelines.xhtml

Table 2: Deprivation at cancer diagnosis/pseudo-diagnosis of subjects identified for inclusion on the case-control study and those for entry into the matched-cohorts distrivuted according to whether the parents participated in the case-control study or not.

		Case-control-study: Subjects targeted for inclusion				Five-year Survivor cohorts			
		Cases		First-choice Controls[1]		Cases		First-choice Controls	
		Parents participated?		Parents participated?		Parents participated?		Parents participated?	
		Yes	No	Yes	No	Yes	No	Yes	No
		N (%)	N (%)	N (%)	N (%)	N (%)	N (%)	N (%)	N (%)
**Total**		3835(100)	595(100)	5526(100)	2137(100)	2819(100)	306(100)	4080(100)	1531(100)
**Deprivation**									
**Affluent**	**1**	774(20.2)	78(13.1)	1249(22.6)	252(11.8)	576(20.4)	37(12.1)	915(22.4)	179(11.7)
	**2**	823(21.5)	90(15.1)	1181(21.4)	313(14.7)	618(21.9)	48(15.7)	873(21.4)	240(15.7)
	**3**	773(20.2)	79(13.3)	1156(20.9)	357(16.7)	579(20.5)	41(13.4)	839(20.6)	241(15.7)
	**4**	755(19.7)	136(22.9)	1051(19.0)	463(21.7)	531(18.8)	73(23.9)	798(19.6)	343(22.4)
**Deprived**	**5**	710(18.5)	169(28.4)	889(16.1)	698(32.7)	515(18.3)	89(29.1)	655(16.0)	485(31.7)
**Not known**		-	43(7.2)	-	54(2.5)	-	18(5.9)	-	43(0.8)
		χ^2=64.8, p<0.001		χ^2=354.5, p<0.001		χ^2=43.3, p<0.001		χ^2=238.0, p<0.001	

[1]Controls were not selected for 595 cases (13.4%) whose parent(s) were not interviewed; and only one first-choice control was identified for 7 of the remaining 3835 cases.

Follow-up

The fact that basic details (matching variables, postcode, NHS number) of all cases and controls were registered and retained, regardless of the families' participation, enabled the conversion of the study into a matched cohort that could track all registered cases (n=4430) and their corresponding first-choice controls (n=7663) who were targeted for interview (**Table 1**; first two columns). In addition to the targeted case- and control-populations, replacement controls are being tracked, enabling the study to also make comparisons between the 3835 cases (**Table 2**, column 1) and 7621 controls (5526 first-choice (**Table 2**, column 3) and 2095 replacement controls) whose parents participated.

Tracking all of registered cases and controls, the UKCCS now operates on a legal basis that permits follow-up information to be obtained from NHS administrative healthcare records without explicit consent, enabling all cases and their corresponding controls to be "flagged" at the national level and tracked via linkage to nationwide information on deaths, cancer registrations, and hospital episode data (inpatient day and overnight admissions; outpatient appointments and visits; accident and emergency presentations; and maternity admissions). Importantly, in line with current ethical and governance requirements, the matched-cohorts are now pseudonymized; NHS identifiers are no longer held by the study but by the national administrative bodies supplying linked data (NHS England, Public Health Scotland, NHS Central Register Scotland, and the Secured Anonymised Information Linkage Databank).

Beginning with the numbers registered in the original case-control study, the flow diagram presented in **Figure 1** gives the numbers of cases and controls who survived five years or more, followed by the numbers who were successfully linked to national administrative data sources**.** Cases enter the survivors' cohort exactly five years after their cancer was first diagnosed, and controls five years after their corresponding case's cancer was first diagnosed (pseudo-diagnosis

For peer review only

date). Controls who emigrated, died or were lost to follow-up before five years had elapsed are not included in the survivor's cohort, nor are controls who were matched to cases who died within five years of their diagnosis; since, where applicable, their follow-up was truncated when their matched case died (**Figure 1**; bottom row). All those who were targeted for interview in the original case-control study are shown on the left-side of **Figure 1**, and all those whose parents were interviewed on the right-side; the total number of individuals that are being followed is shown in the yellow boxes in the centre of the figure.

Figure 1

With respect to the available data, the time periods currently covered by the various linked healthcare datasets, which are updated on an annual basis are shown in **Supplematry Figure 2;** and the key data domains and fields are listed in **Box 1.**

Box 1: National administrative data available for comparative analysis: United Kingdom Childhood Cancer Study (UKCCS)

Source of data		Description of fields
Socio-demographic characteristics		Sex; year of birth; date of diagnosis/pseudo-diagnosis; indicators of deprivation at diagnosis/pseudo-diagnosis (Postcode sources; birth certificates, FHSA/HB[a] registers and parental interviews, HES[b])
Death notifications		Date and causes of death (ICD-10[c])
Emigrations		Date of embarkation
Cancer Registrations		Date of diagnosis; date of registration; topography (ICD revisions 7-10); morphology (ICD-O[d] revisions 1-3)
Secondary Care Data (Hospital Activity – HES[b]) Data		
	Inpatient and Day Cases	Date of admission; date of discharge; dates and types of procedures (OPCS-4[e], maximum 24); conditions at discharge (ICD-10[c], maximum 20); consultant specialties involved; source of referral; discharge destination
	Outpatient	Date of appointment and attendance flag; dates of procedures (maximum 24); consultant specialties involved
	Accident & Emergency	Date and reason for attendance; investigations and treatments; source of referral
	Maternity Inpatient and Day Cases	Date of admission to obstetrics or midwifery; date of discharge; conditions at discharge (ICD-10[c] maximum 20); age at birth; delivery method; babies' birthweights

[a]FHSA/HB: Family Health Strategic Authority or Health Board
[b]HES: Hospital Episode Statistics in England, or equivalently, Scottish Morbidity Record (SMR) in Scotland, and the Patient Episode Database for Wales (PEDW).
[c]ICD: International Classification of Diseases.
[d]ICD-O: International Classification of Diseases for Oncology.
[e]OPCS-4: Office of Population Censuses and Surveys Classification of Interventions and Procedures version 4.

For peer review only - http://bmjopen.bmj.com/site/about/guidelines.xhtml

Patient and public involvement and Engagement (PPIE)

The original case-control study was established over 30 years ago, at a time when lay-involvement in research was largely absent, and so did not benefit from formal links with patient/user groups. Members of the CCLG (Children's Cancer and Leukaemia Group, previously the UK Children's Cancer Study Group) were, however, fully involved throughout; and the study website (www.UKCCS.org) contains information about ongoing research activities and fair processing. The transformation of the study to focus on late effects among long-term childhood cancer survivors means that we can now move forwards to enable patients and the public to work in partnership with researchers and clinical staff to co-develop and deliver the research, disseminate findings, and determine the direction of future embedded studies. This input will ensure data are utilized in a way that secures maximize impact, improves long-term care, and promotes effective resource planning and management in the future.

Characteristics of individuals in the study

Detailed diagnostic and treatment information was obtained from multiple sources, including diagnosing/treatment centres, national treatment trials (where they existed), hospital records and reference laboratories. The diagnostic distribution, coded to the International Classification of Childhood Cancer 3rd edition (ICCC-3), as well as information on age, sex and interview status, is shown in **Table 3**. As expected, given the increasing emphasis on leukaemia in the later years of the case-control study's data collection phase, the final diagnostic distribution was weighted towards leukaemia. Variations with parental interview status are also evident; the non-interviewed group containing proportionally more CNS tumours (ICCC III) and other cancers (ICCC XI and XII). Among all registered cases, 1195 (27.0%) died within the first five years, and while in total, over 70% of the cases were alive at five years after diagnosis, the proportions within each ICCC-3 group varied with cancer (**Table 4**).

For peer review only - http://bmjopen.bmj.com/site/about/guidelines.xhtml

Table 3: Diagnostic frequencies, median ages at diagnosis, and parental interview status of children (0-14 years) registered in the United Kingdom Childhood Cancer Study (UKCCS)

Diagnosis (ICCC group)	Total N (%)	Males (%)	Age, median (interquartile range)	Interviewed Yes N (%)	Interviewed No N (%)
All cancers	4430(100.0)	55.7	5.1(2.7-9.7)	3835(100.0)	595(100.0)
Leukaemia (I)	1911(43.1)	55.8	4.5(2.8-8.0)	1734(45.2)	177(29.7)
Acute lymphoblastic leukaemia (Ia)	1580(35.7)	55.8	4.4(2.9-7.6)	1462(38.1)	118(19.8)
Acute myeloid leukaemia (Ib)	294(6.6)	53.7	5.4(1.8-11.2)	248(6.5)	46(7.7)
Lymphomas & reticuloendothelial (II)	413(9.3)	72.9	9.9(5.8-12.9)	357(9.3)	56(9.4)
Hodgkin lymphoma (IIa)	132(3.0)	71.2	11.6(7.9-13.5)	117(3.0)	15(2.5)
Non-Hodgkin lymphoma (IIb)	258(5.8)	72.9	9.4(5.2-12.0)	226(5.9)	32(5.4)
CNS, intracranial & intraspinal (III)	848(19.1)	50.1	6.5(3.3-10.1)	686(17.9)	162(27.2)
Ependymoma and choroid plexus (IIIa)	101(2.3)	58.4	3.4(1.4-9.0)	90(2.3)	11(1.8)
Astrocytoma (IIIb)	372(8.4)	44.1	6.9(4.0-10.5)	303(7.9)	69(11.6)
Intracranial & intraspinal embryonal (IIIc)	189(4.3)	55.6	6.0(2.7-8.8)	162(4.2)	27(4.5)
Neuroblastoma & other peripheral neural (IV)	222(5.0)	59.5	1.8(0.7-3.7)	188(4.9)	34(5.7)
Retinoblastoma (V)	112(2.5)	55.4	1.4(0.7-2.7)	87(2.3)	25(4.2)
Renal (VI)	221(5.0)	51.6	3.2(1.8-5.2)	199(5.2)	22(3.7)
Hepatic (VII)	36(0.8)	58.3	1.5(0.9-3.3)	32(0.8)	4(0.7)
Bone (VIII)	121(2.7)	53.7	11.2(8.9-13.5)	106(2.8)	15(2.5)
Soft tissue & other sarcomas (IX)	266(6.0)	54.9	5.7(2.8-10.6)	233(6.1)	33(5.5)
Germ cell & gonadal (X)	114(2.6)	54.4	6.9(1.4-12.3)	98(2.5)	16(2.7)
Other cancers (XI and XII)	99(2.2)	43.4	10.6(6.6-13.5)	62(1.6)	37(6.2)

For peer review only

For peer review only - http://bmjopen.bmj.com/site/about/guidelines.xhtml

Table 4: Number of children in the case-control study, numbers alive five years after diagnosis, and current duration of follow-up (July 2020 England and Wales; June 2022 Scotland) by diagnosis (ICCC-3): UKCCS

Diagnosis (ICCC group)	Total	Alive 5-years after diagnosis[1]		Total Person-Years[2]	
		N	%	Median (IQR)	Total
**Total Cases**	4430	3212	72.5	25.3(3.5-27.1)	78,623
Leukaemias (I)	1911	1448	75.8	24.7(5.2-26.7)	34,423
Acute lymphoblastic leukaemia (Ia)	1580	1275	80.7	24.9(9.0-26.9)	30,206
Acute myeloid leukaemia (Ib)	294	161	54.8	19.4(0.9-25.8)	3,913
Lymphomas & reticuloendothelial (II)	413	344	83.3	26.1(20.4-27.5)	8,434
Hodgkin lymphoma (IIa)	132	127	96.2	27.3(26.1-28.2)	3,194
Non-Hodgkin lymphoma (IIb)	258	203	78.7	25.4(12.9-27.1)	4,880
CNS, intracranial and intraspinal (III)	848	534	63.0	23.3(1.3-26.9)	13,021
Ependymoma and choroid plexus (IIIa)	101	62	61.4	9.2(1.9-26.5)	1,359
Astrocytoma (IIIb)	372	275	73.9	26.2(3.6-27.5)	6,894
Intracranial embryonal (IIIc)	189	84	44.4	3.0(0.8-26.1)	2,050
Neuroblastoma and other peripheral neural (IV)	222	121	54.5	14.7(1.3-26.8)	3,109
Retinoblastoma (V)	112	108	96.4	26.8(26.0-27.5)	2,769
Renal (VI)	221	173	78.3	26.2(19.4-27.4)	4,477
Hepatic (VII)	36	27	75.0	25.8(2.6-26.9)	674
Bone (VIII)	121	70	57.9	20.0(1.9-26.9)	1,799
Soft tissue and other sarcomas (IX)	266	167	62.8	25.2(1.8-27.4)	4,205
Germ cell and gonadal (X)	114	92	80.7	26.5(18.7-27.5)	2,359
Other cancers (XI & XII)	99	70	70.7	26.5(2.4-27.7)	1,793
**Total Controls**	9758	9703	99.4	26.5(25.2-27.6)	244,061
All first choice	7663	7601	99.2	26.6(25.2-27.6)	191,484
Replacements (parents interviewed)	2095	2091	99.8	26.5(25.1-27.5)	52,577

[1]97.7% of cases and 97.1% of controls have been traced and have administrative data.
[2]Person-years are calculated for the total number of cases from diagnosis to end of follow-up, and for controls from their pseudo-diagnosis date, i.e. the date they were the same age as their matched case was at diagnosis

FINDINGS TO DATE

The UKCCS provides a valuable resource within which to examine both the potential causes and long-term health consequences of childhood cancer. Thus far, over 90 peer-reviewed articles containing UKCCS data have been published, and a full list is on the study website (www.UKCCS.org). Assembling data from multiple sources, key reports include ALL genome-wide association studies (GWAS)[14,20], and those examining the potential aetiological roles of a wide range of biological (e.g. infections/markers of infectious exposure)[21–23], physical (e.g. ionizing/non-ionizing radiation)[12,13,24,25] and chemical (e.g. prescription drugs/smoking) agents acting on parents before the child's conception and the child during pregnancy/early life[10,26,27], as well as relationships with other illnesses (e.g. allergy) and birth characteristics (e.g. birthweight)[21,28,29]. Importantly, as noted above, the UKCCS accessed medical records to help answer key questions relating to prescription drugs, illness histories and co-morbidities; such contemporaneously recorded information, which by its nature is free from differential recall bias, often producing very different results from that obtained by self-report[11,22,30,31].

For peer review only - http://bmjopen.bmj.com/site/about/guidelines.xhtml

With respect to the longer-term health and healthcare needs of childhood cancer survivors, the conversion of the UKCCS into a matched cohort is now yielding important results about the morbidity and mortality of childhood cancer survivors. Early findings based on the case-population alone highlighted survival variations with age and sex across a range of subtypes[15], and for acute lymphoblastic leukaemia, survival associations with area-based measures of deprivation at the time of diagnosis were also noted[16]. With additional years of follow-up, we are now able to report a 25-year survival of 65.3% for all cancers combined (95% confidence interval 63.8-66.7). More recently, we have begun examining secondary care hospital activity patterns, comparing findings in the case-population to those in the control-population over the first 25 years. Adding to excess risks of death and cancer, our first report demonstrated that survivors of childhood acute lymphoblastic leukaemia (ALL) experienced excess outpatient and inpatient activity across their teenage and young adult years; which was not related to routine follow-up monitoring. Indeed, with hospital activity being higher than expected in specialties covering most organ and tissue systems, survivors were more than twice as likely to fall under the care of endocrinology, cardiology, respiratory medicine, ophthalmology, neurology and/or gastroenterology specialists[17]. Furthermore, these differences showed no signs of diminishing in the first 25 years of follow-up, underscoring the need to take prior cancer drug and/or radiation treatment into account when interpreting seemingly unrelated symptoms in later life.

STRENGTHS AND LIMITATIONS

Covering England, Scotland and Wales, findings from this population-based age- and sex- matched cohort study are based on routinely compiled linked health data, not self-report. With minimal selection bias and loss to follow-up,the matched-cohort design allows more granular analyses to be performed than is possible in case-cohorts that rely solely on general population rates, as has been done in other UK-based studies[32–35]. For example, at the point of cohort entry the individually age- and sex-matched control-population has a similar deprivation distribution to the case-population, providing a robust baseline against which to evaluate the potential impact of deprivation across the life-course. In addition, because the UKCCS cohorts can be divided into those whose families participated in the original case-control study and those who did not, the study is well-placed to investigate the impact of selection bias; an analysis which most cohorts cannot undertake, largely because they were not designed to do so, most being either record-based (as on the left side of **Figure 1**) [36–39]**,** or interview-based (as on the right-side of **Figure 1**) [40,41]. Thus far, with follow-up extending to over 25 years since diagnosis, we have examined health from childhood through TYA and early adulthood, and both cohorts will continue to be tracked as they age. With respect to limitations, lack of primary care data and information from psychosocial services are obvious deficiencies that currently affects all UK record-linkage studies of the type described here [42–44]. Analyses are also constrained by the fact that national administrative healthcare data are primarily collected for administrative and clinical purposes, and not for research.

COLLABORATION

To protect privacy and confidentiality, approval for the linkage of the UKCCS to health data is provided under strict conditions for the storage, retention and use of the data. The current approvals permit storage of the data at one site (University of York) for use by individually named researchers. We encourage interested parties to contact us to discuss potential analyses. Reasonable requests for data access can be submitted to the study Principal Investigator, ER (eve.roman@york.ac.uk), for review by the UKCCS investigator team. The PI will contact the relevant agencies to explore the feasibility of data sharing subject to ethical and data access agreements; should data sharing be agreeable with the agencies, approval for data access and

For peer review only - http://bmjopen.bmj.com/site/about/guidelines.xhtml

specific analyses will be required from the appropriate ethics committees and data sharing agreements put in place.

DATA SHARING

Reasonable requests for data sharing will be considered by the authors.

Ethics approval

The United Kingdom Childhood Cancer Study (UKCCS) has ethics approval from the Yorkshire & the Humber-Leeds West Research Ethics Committee (reference 18/YH/0135) and exemption from Section 251 of the Health & Social Care Act (reference 18/CAG/0066).

Author contributions

ER contributed to the design of the original case-control study, initiated the matched cohort, and is the guarantor. SK and JS have also been involved with the study since its inception. EK was involved in initiating the matched cohort and leads on cohort data management. AB, EK, JS, and AS have conducted many of the analyses. SK is the clinical lead, and DH and RS have extensive experience of working with patients and carers to deliver on PPIE. All authors have contributed to this report.

Funding

The UKCCS work reported on here was previously supported by Blood Cancer UK grant number 15037, and is jointly now supported by Blood Cancer UK and Cancer Research UK (grant number 29685). AB is supported by a fellowship from the Fondation ARC pour la Recherche sur le Cancer (PDF20190508759). The funders had no role in considering the study design or in the collection, analysis, interpretation of data, writing of the manuscript, or the decision to submit the article for publication.

Conflicts of interest

None of the authors have any conflicts of interest

References

1 Gatta G, Botta L, Rossi S, *et al.* Childhood cancer survival in Europe 1999-2007: results of EUROCARE-5--a population-based study. *Lancet Oncol* 2014;**15**:35–47. doi:10.1016/S1470-2045(13)70548-5

2 American Cancer Society. Cancer Facts & Figures 2015. Atlanta, GA, USA: : American Cancer Society, Inc. 2015. https://www.cancer.org/research/cancer-facts-statistics/all-cancer-facts-figures/cancer-facts-figures-2015.html (accessed 2 Dec 2022).

3 The European Society for Paediatric Oncology. SIOP Europe Strategic Plan. Update (2021-2026). A European Cancer Plan for Children and Adolescents. Brussels, Belgium: : SIOP Europe 2021. https://siope.eu/european-strategic-plan/

4 Kebudi R, Özdemir GN. Second malignant neoplasms in childhood cancer survivors. *Curr Pediatr Rev* 2017;**13**:34–41.

5 Fidler MM, Reulen RC, Henson KE, *et al.* Population-Based Long-Term Cardiac-Specific Mortality Among 34,489 Five-Year Survivors of Childhood Cancer in Great Britain. *Circulation* 2017;**135**:951–63. doi:10.1161/CIRCULATIONAHA.116.024811

For peer review only - http://bmjopen.bmj.com/site/about/guidelines.xhtml

6 Dietz AC, Chen Y, Yasui Y, *et al.* Risk and impact of pulmonary complications in survivors of childhood cancer: A report from the Childhood Cancer Survivor Study. *Cancer* 2016;**122**:3687–96. doi:10.1002/cncr.30200

7 Overbeek A, van den Berg MH, van Leeuwen FE, *et al.* Chemotherapy-related late adverse effects on ovarian function in female survivors of childhood and young adult cancer: A systematic review. *Cancer Treat Rev* 2017;**53**:10–24. doi:10.1016/j.ctrv.2016.11.006

8 UK Childhood Cancer Study Investigators. The United Kingdom Childhood Cancer Study: objectives, materials and methods. *Br J Cancer* 2000;**82**:1073–102. doi:10.1054/bjoc.1999.1045

9 Day N, Skinner J, Roman E, *et al.* Exposure to power-frequency magnetic fields and the risk of childhood cancer. UK Childhood Cancer Study Investigators. *Lancet* 1999;**354**:1925–31.

10 Roman E, Simpson J, Ansell P, *et al.* Perinatal and reproductive factors: a report on haematological malignancies from the UKCCS. *Eur J Cancer* 2005;**41**:749–59. doi:10.1016/j.ejca.2004.11.006

11 Roman E, Simpson J, Ansell P, *et al.* Infectious proxies and childhood leukaemia: Findings from the United Kingdom Childhood Cancer Study (UKCCS). *Blood Cells, Molecules, and Diseases* 2009;**42**:126–8. doi:10.1016/j.bcmd.2008.10.007

12 UK Childhood Cancer Study Investigators. The United Kingdom Childhood Cancer Study of exposure to domestic sources of ionising radiation: 1: radon gas. *Br J Cancer* 2002;**86**:1721–6. doi:10.1038/sj.bjc.6600276

13 UK Childhood Cancer Study Investigators. The United Kingdom Childhood Cancer Study of exposure to domestic sources of ionising radiation: 2: gamma radiation. *Br J Cancer* 2002;**86**:1727–31. doi:10.1038/sj.bjc.6600277

14 Papaemmanuil E, Hosking FJ, Vijayakrishnan J, *et al.* Loci on 7p12.2, 10q21.2 and 14q11.2 are associated with risk of childhood acute lymphoblastic leukemia. *Nat Genet* 2009;**41**:1006–10. doi:10.1038/ng.430

15 Johnston WT, Lightfoot TJ, Simpson J, *et al.* Childhood cancer survival: a report from the United Kingdom Childhood Cancer Study. *Cancer Epidemiol* 2010;**34**:659–66. doi:10.1016/j.canep.2010.06.020

16 Lightfoot TJ, Johnston WT, Simpson J, *et al.* Survival from childhood acute lymphoblastic leukaemia: the impact of social inequality in the United Kingdom. *Eur J Cancer* 2012;**48**:263–9. doi:10.1016/j.ejca.2011.10.007

17 Kane E, Kinsey S, Bonaventure A, *et al.* Excess morbidity and mortality among survivors of childhood acute lymphoblastic leukaemia: 25 years of follow-up from the United Kingdom Childhood Cancer Study (UKCCS) population-based matched cohort. *BMJ Open* 2022;**12**:e056216. doi:10.1136/bmjopen-2021-056216

18 Smith A, Roman E, Simpson J, *et al.* Childhood leukaemia and socioeconomic status: fact or artefact? A report from the United Kingdom childhood cancer study (UKCCS). *International Journal of Epidemiology* 2006;**35**:1504–13. doi:10.1093/ije/dyl193

19 Stiller CA, Allen MB, Eatock EM. Childhood cancer in Britain: the National Registry of Childhood Tumours and incidence rates 1978-1987. *Eur J Cancer* 1995;**31A**:2028–34.

For peer review only - http://bmjopen.bmj.com/site/about/guidelines.xhtml

20 Vijayakrishnan J, Kumar R, Henrion MYR, *et al.* A genome-wide association study identifies risk loci for childhood acute lymphoblastic leukemia at 10q26.13 and 12q23.1. *Leukemia* 2017;**31**:573–9. doi:10.1038/leu.2016.271

21 Roman E, Simpson J, Ansell P, *et al.* Childhood Acute Lymphoblastic Leukemia and Infections in the First Year of Life: A Report from the United Kingdom Childhood Cancer Study. *American Journal of Epidemiology* 2007;**165**:496–504. doi:10.1093/aje/kwk039

22 Simpson J, Smith A, Ansell P, *et al.* Childhood leukaemia and infectious exposure: A report from the United Kingdom Childhood Cancer Study (UKCCS). *European Journal of Cancer* 2007;**43**:2396–403. doi:10.1016/j.ejca.2007.07.027

23 Crouch S, Lightfoot T, Simpson J, *et al.* Infectious illness in children subsequently diagnosed with acute lymphoblastic leukemia: modeling the trends from birth to diagnosis. *American journal of epidemiology* 2012;**176**:402–8.

24 Ahlbom A, Day N, Feychting M, *et al.* A pooled analysis of magnetic fields and childhood leukaemia. *Br J Cancer* 2000;**83**:692–8. doi:10.1054/bjoc.2000.1376

25 Skinner J, Mee TJ, Blackwell RP, *et al.* Exposure to power frequency electric fields and the risk of childhood cancer in the UK. *Br J Cancer* 2002;**87**:1257–66. doi:10.1038/sj.bjc.6600602

26 Roman E, Fear NT, Ansell P, *et al.* Vitamin K and childhood cancer: analysis of individual patient data from six case–control studies. *British Journal of Cancer* 2002;**86**:63–9.

27 Bonaventure A, Simpson J, Ansell P, *et al.* Prescription drug use during pregnancy and risk of childhood cancer – Is there an association? *Cancer Epidemiology* 2015;**39**:73–8. doi:10.1016/j.canep.2014.10.008

28 Bonaventure A, Simpson J, Ansell P, *et al.* Paediatric acute lymphoblastic leukaemia and caesarean section: A report from the United Kingdom Childhood Cancer Study (UKCCS). *Paediatric and Perinatal Epidemiology* 2020;**34**:344–9. doi:10.1111/ppe.12662

29 Milne E, Greenop KR, Metayer C, *et al.* Fetal growth and childhood acute lymphoblastic leukemia: Findings from the childhood leukemia international consortium. *International Journal of Cancer* 2013;**133**:2968–79. doi:10.1002/ijc.28314

30 Smith A, Roman E, Simpson J. Childhood leukaemia and socioeconomic status. *International Journal of Epidemiology* 2007;**36**:1156. doi:10.1093/ije/dym166

31 Bonaventure A, Kane E, Simpson J, *et al.* Maternal infections and medications in pregnancy: how does self-report compare to medical records in childhood cancer case–control studies? *International Journal of Epidemiology* 2023;:dyad019. doi:10.1093/ije/dyad019

32 van Laar M, Feltbower RG, Gale CP, *et al.* Cardiovascular sequelae in long-term survivors of young peoples' cancer: a linked cohort study. *Br J Cancer* 2014;**110**:1338–41. doi:10.1038/bjc.2014.37

33 Reulen RC, Bright CJ, Winter DL, *et al.* Pregnancy and Labor Complications in Female Survivors of Childhood Cancer: The British Childhood Cancer Survivor Study. *J Natl Cancer Inst* 2017;**109**. doi:10.1093/jnci/djx056

For peer review only

For peer review only - http://bmjopen.bmj.com/site/about/guidelines.xhtml

34 Smith L, Glaser AW, Peckham D, *et al.* Respiratory morbidity in young people surviving cancer: Population-based study of hospital admissions, treatment-related risk factors and subsequent mortality. *Int J Cancer* 2019;**145**:20–8. doi:10.1002/ijc.32066

35 Reulen RC, Guha J, Bright CJ, *et al.* Risk of cerebrovascular disease among 13 457 five-year survivors of childhood cancer: A population-based cohort study. *International Journal of Cancer* 2021;**148**:572–83. doi:10.1002/ijc.33218

36 McBride ML, Rogers PC, Sheps SB, *et al.* Childhood, adolescent, and young adult cancer survivors research program of British Columbia: objectives, study design, and cohort characteristics. *Pediatr Blood Cancer* 2010;**55**:324–30. doi:10.1002/pbc.22476

37 Asdahl PH, Winther JF, Bonnesen TG, *et al.* The Adult Life After Childhood Cancer in Scandinavia (ALiCCS) Study: Design and Characteristics. *Pediatr Blood Cancer* 2015;**62**:2204–10. doi:10.1002/pbc.25661

38 Kirchhoff AC, Fluchel MN, Wright J, *et al.* Risk of Hospitalization for Survivors of Childhood and Adolescent Cancer. *Cancer Epidemiol Biomarkers Prev* Published Online First: 12 June 2014. doi:10.1158/1055-9965.EPI-13-1090

39 Streefkerk N, Tissing WJE, Korevaar JC, *et al.* A detailed insight in the high risks of hospitalizations in long-term childhood cancer survivors-A Dutch LATER linkage study. *PLoS ONE* 2020;**15**:e0232708. doi:10.1371/journal.pone.0232708

40 Robison LL, Mertens AC, Boice JD, *et al.* Study design and cohort characteristics of the Childhood Cancer Survivor Study: a multi-institutional collaborative project. *Med Pediatr Oncol* 2002;**38**:229–39. doi:10.1002/mpo.1316

41 Hawkins MM, Lancashire ER, Winter DL, *et al.* The British Childhood Cancer Survivor Study: Objectives, methods, population structure, response rates and initial descriptive information. *Pediatr Blood Cancer* 2008;**50**:1018–25. doi:10.1002/pbc.21335

42 Chow EJ, Ness KK, Armstrong GT, *et al.* Current and coming challenges in the management of the survivorship population. *Semin Oncol* 2020;**47**:23–39. doi:10.1053/j.seminoncol.2020.02.007

43 Andrés-Jensen L, Skipper MT, Mielke Christensen K, *et al.* National, clinical cohort study of late effects among survivors of acute lymphoblastic leukaemia: the ALL-STAR study protocol. *BMJ Open* 2021;**11**. doi:10.1136/bmjopen-2020-045543

44 van Erp LME, Maurice-Stam H, Kremer LCM, *et al.* A vulnerable age group: the impact of cancer on the psychosocial well-being of young adult childhood cancer survivors. *Support Care Cancer* 2021;**29**:4751–61. doi:10.1007/s00520-021-06009-y

For peer review only

For peer review only - http://bmjopen.bmj.com/site/about/guidelines.xhtml

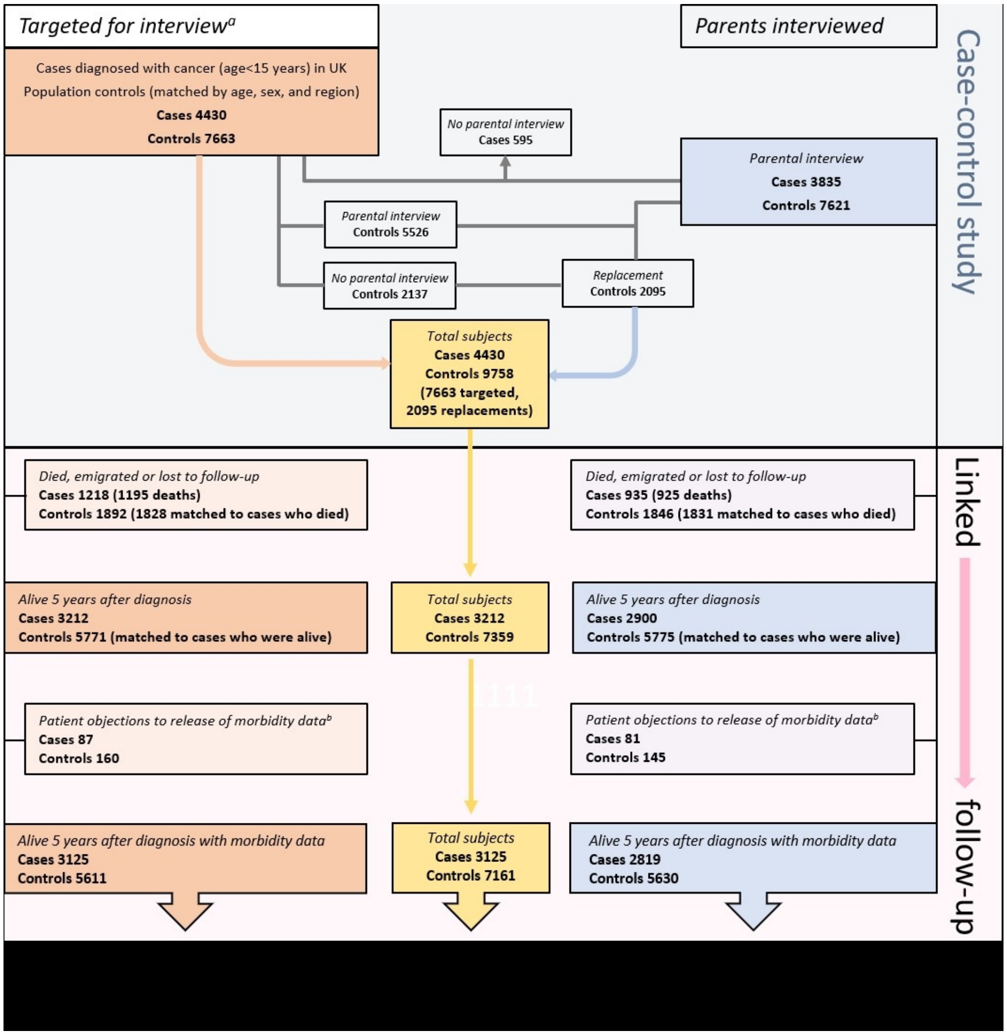

Figure 1: UKCCS subjects distributed by parental interviewed status (total targeted and total interviewed) and follow-up status; the numbers of 5-year survivors with linked health data that are being followed are presented in the bottom row.

180x184mm (150 x 150 DPI)

Supplementary Figure 1

United Kingdom Childhood Cancer Study (UKCCS) administrative areas

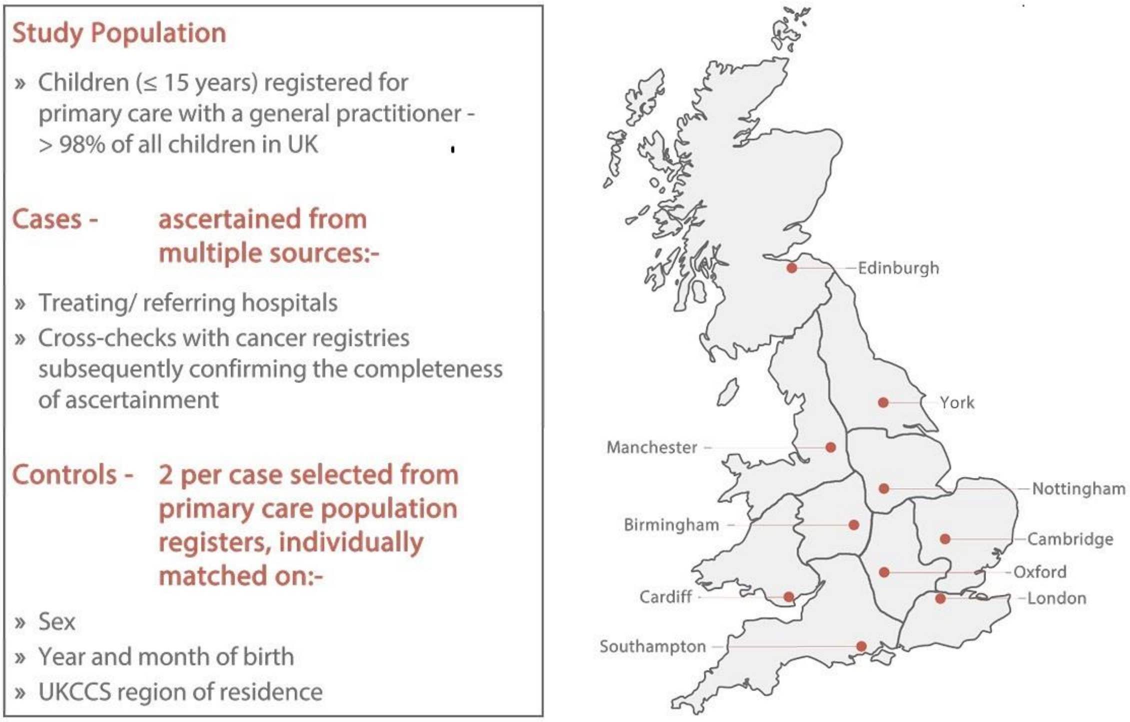

eview only

Supplementary Figure 2

United Kingdom Childhood Cancer Study (UKCCS) linkage to national administrative datasets

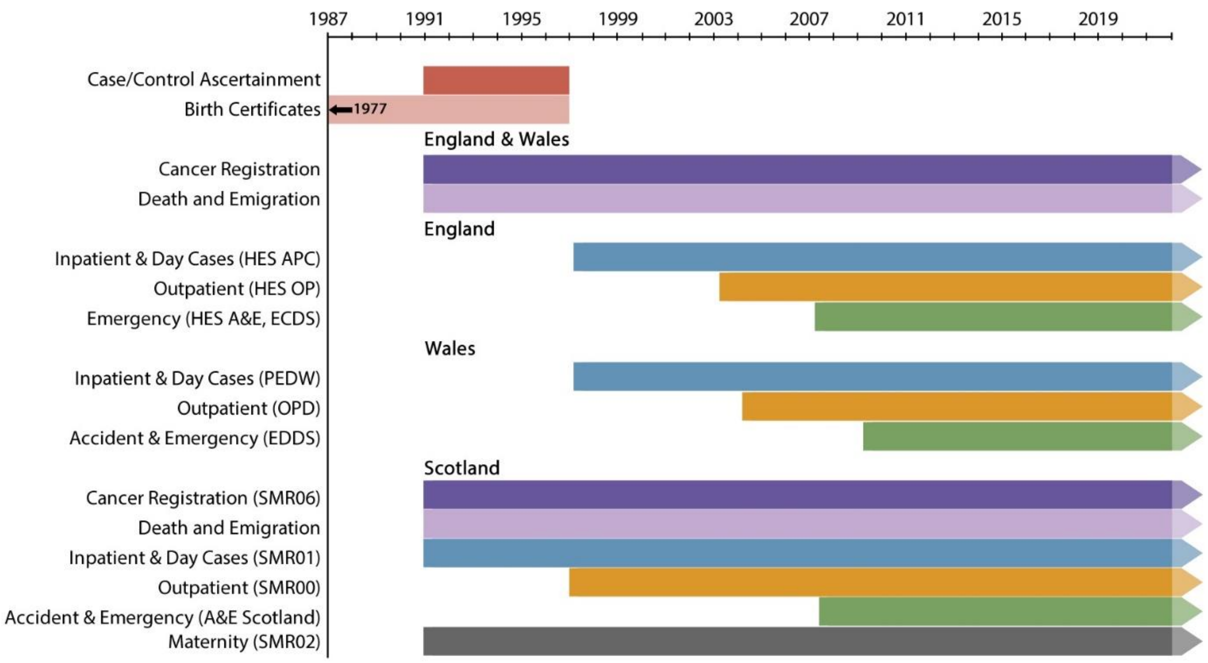

UKCCS: United Kingdom Childhood Cancer Study; HES: Hospital Episode Statistics; APC: Admitted Patient Care; OP: Outpatients; ECDS: Emergency Care Dataset; PEDW: Patient Episode Data for Wales; OPD: Outpatient Data (Wales); EDD: Emergency Department Dataset (Wales); SMR: Scottish Morbidity Record

STROBE 2007 (v4) Statement—Checklist of items that should be included in reports of *cohort studies*

Section/Topic	Item #	Recommendation	Reported on page #
**Title and abstract**	1	(*a*) Indicate the study's design with a commonly used term in the title or the abstract	1-2
		(*b*) Provide in the abstract an informative and balanced summary of what was done and what was found	2
**Introduction**			
Background/rationale	2	Explain the scientific background and rationale for the investigation being reported	2-4
Objectives	3	State specific objectives, including any prespecified hypotheses	2
**Methods**			
Study design	4	Present key elements of study design early in the paper	2
Setting	5	Describe the setting, locations, and relevant dates, including periods of recruitment, exposure, follow-up, and data collection	2-5
Participants	6	(*a*) Give the eligibility criteria, and the sources and methods of selection of participants. Describe methods of follow-up	3-4
		(*b*) For matched studies, give matching criteria and number of exposed and unexposed	3
Variables	7	Clearly define all outcomes, exposures, predictors, potential confounders, and effect modifiers. Give diagnostic criteria, if applicable	3-6
Data sources/ measurement	8*	For each variable of interest, give sources of data and details of methods of assessment (measurement). Describe comparability of assessment methods if there is more than one group	3-6
Bias	9	Describe any efforts to address potential sources of bias	3-6
Study size	10	Explain how the study size was arrived at	3-8
Quantitative variables	11	Explain how quantitative variables were handled in the analyses. If applicable, describe which groupings were chosen and why	3-7
Statistical methods	12	(*a*) Describe all statistical methods, including those used to control for confounding	3-6
		(*b*) Describe any methods used to examine subgroups and interactions	5
		(*c*) Explain how missing data were addressed	5
		(*d*) If applicable, explain how loss to follow-up was addressed	5
		(*e*) Describe any sensitivity analyses	NA
**Results**			

For peer review only

Participants	13*	(a) Report numbers of individuals at each stage of study—eg numbers potentially eligible, examined for eligibility, confirmed eligible, included in the study, completing follow-up, and analysed	5
		(b) Give reasons for non-participation at each stage	3-5
		(c) Consider use of a flow diagram	3
Descriptive data	14*	(a) Give characteristics of study participants (eg demographic, clinical, social) and information on exposures and potential confounders	4,7,8
		(b) Indicate number of participants with missing data for each variable of interest	8
		(c) Summarise follow-up time (eg, average and total amount)	8
Outcome data	15*	Report numbers of outcome events or summary measures over time	8
Main results	16	(*a*) Give unadjusted estimates and, if applicable, confounder-adjusted estimates and their precision (eg, 95% confidence interval). Make clear which confounders were adjusted for and why they were included	NA
		(*b*) Report category boundaries when continuous variables were categorized	NA
		(*c*) If relevant, consider translating estimates of relative risk into absolute risk for a meaningful time period	NA
Other analyses	17	Report other analyses done—eg analyses of subgroups and interactions, and sensitivity analyses	NA
**Discussion**			
Key results	18	Summarise key results with reference to study objectives	8-9
**Limitations**			
Interpretation	20	Give a cautious overall interpretation of results considering objectives, limitations, multiplicity of analyses, results from similar studies, and other relevant evidence	8-9
Generalisability	21	Discuss the generalisability (external validity) of the study results	9
**Other information**			
Funding	22	Give the source of funding and the role of the funders for the present study and, if applicable, for the original study on which the present article is based	10

*Give information separately for cases and controls in case-control studies and, if applicable, for exposed and unexposed groups in cohort and cross-sectional studies.

Note: An Explanation and Elaboration article discusses each checklist item and gives methodological background and published examples of transparent reporting. The STROBE checklist is best used in conjunction with this article (freely available on the Web sites of PLoS Medicine at http://www.plosmedicine.org/, Annals of Internal Medicine at http://www.annals.org/, and Epidemiology at http://www.epidem.com/). Information on the STROBE Initiative is available at www.strobe-statement.org.

For peer review only

Cohort Profile: The United Kingdom Childhood Cancer Study (UKCCS): a UK-wide population-based study examining the health of cancer survivors

Journal:	*BMJ Open*
Manuscript ID	bmjopen-2023-073712.R2
Article Type:	Cohort profile
Date Submitted by the Author:	19-Oct-2023
Complete List of Authors:	Roman, Eve; University of York, Health Sciences<br>Kane, Eleanor; University of York, Health Sciences<br>Smith, Alexandra; University of York, Health Sciences<br>Howell, Debra; University of York, Health Sciences<br>Sheridan, Rebecca; University of York,<br>Simpson, Jill; University of York<br>Bonaventure, Audrey; INSERM<br>Kinsey, Sally; Leeds Children's Hospital, Paediatric Haematology
**Primary Subject Heading**:	Public health
Secondary Subject Heading:	Epidemiology
Keywords:	Leukaemia < HAEMATOLOGY, Lymphoma < ONCOLOGY, EPIDEMIOLOGY, PUBLIC HEALTH, Quality of Life, Risk Factors

SCHOLARONE™ Manuscripts

I, the Submitting Author has the right to grant and does grant on behalf of all authors of the Work (as defined in the below author licence), an exclusive licence and/or a non-exclusive licence for contributions from authors who are: i) UK Crown employees; ii) where BMJ has agreed a CC-BY licence shall apply, and/or iii) in accordance with the terms applicable for US Federal Government officers or employees acting as part of their official duties; on a worldwide, perpetual, irrevocable, royalty-free basis to BMJ Publishing Group Ltd ("BMJ") its licensees and where the relevant Journal is co-owned by BMJ to the co-owners of the Journal, to publish the Work in this journal and any other BMJ products and to exploit all rights, as set out in our licence.

The Submitting Author accepts and understands that any supply made under these terms is made by BMJ to the Submitting Author unless you are acting as an employee on behalf of your employer or a postgraduate student of an affiliated institution which is paying any applicable article publishing charge ("APC") for Open Access articles. Where the Submitting Author wishes to make the Work available on an Open Access basis (and intends to pay the relevant APC), the terms of reuse of such Open Access shall be governed by a Creative Commons licence – details of these licences and which Creative Commons licence will apply to this Work are set out in our licence referred to above.

Other than as permitted in any relevant BMJ Author's Self Archiving Policies, I confirm this Work has not been accepted for publication elsewhere, is not being considered for publication elsewhere and does not duplicate material already published. I confirm all authors consent to publication of this Work and authorise the granting of this licence.

For peer review only

Cohort Profile: The United Kingdom Childhood Cancer Study (UKCCS): a UK-wide population-based study examining the health of cancer survivors

Eve Roman[1*]

Eleanor Kane[1]

Alexandra Smith[1]

Debra Howell[1]

Rebecca Sheridan [1]

Jill Simpson[1]

Audrey Bonaventure[2]

Sally Kinsey[1]

[1] Epidemiology and Cancer Statistics Group, Department of Health Sciences, University of York, YO10 5DD, UK

*Corresponding author: eve.roman@york.ac.uk

Word count: Main text = 3766

For peer review only

Abstract

Purpose: The United Kingdom Childhood Cancer Study's (UKCCS's) matched-cohort was established to examine the longer-term morbidity and mortality of individuals previously diagnosed with cancer before 15 years of age; comparing future healthcare patterns in 5-year cancer survivors to baseline activity seen in age- and sex-matched individuals from the general population.

Participants: Predicated on a national childhood cancer case-control study conducted in the early 1990s (4430 cases, 9758 controls) in England, Scotland and Wales, the case-population comprises 3125 cancer survivors (>5 years), and the control-population 7161 age- and sex-matched individuals from the general population who did not have cancer as a child. Participants are now being followed-up via linkage to national administrative healthcare databases (deaths, cancers, and secondary-care hospital activity).

Findings to date: Enabling the creation of cohorts with minimal selection bias and loss to follow-up, the original case-control study registered all newly diagnosed cases of childhood cancer and their corresponding controls, regardless of their family's participation. Early findings based on the registered case-population found marked survival variations with age and sex across subtypes, and differences with deprivation among acute lymphoblastic leukaemia (ALL) survivors. More recently, comparing the health-activity patterns of the case- and control-populations, revealed that survivors of childhood ALL experienced excess outpatient and inpatient activity across their teenage/young adult years. Adding to increased risks of cancer and death and involving most clinical specialties, excesses were not related to routine follow-up monitoring, and showed no signs of diminishing over time.

Future plans: With annual linkage updates, the UKCCS's maturing population-based matched-cohorts provide the foundation for tracking the health of individuals through their lifetime. Comparing the experience of childhood cancer survivors to that of unaffected general-population counterparts, this will include examining subsequent morbidity and mortality, secondary care hospital activity, and the impact of deprivation on longer-term outcomes.

Strengths and limitations of this study (max 5 bullet points)

- Covering England, Scotland and Wales, findings from this national study are based on routinely compiled linked health data, not self-report.
- Regardless of whether or not their families participated in the original case-control study, all children are included in the matched cohort, enabling the investigation of selection bias.
- With minimal selection bias and loss to follow-up, the individually age- and sex-matched control-population has a similar deprivation distribution to the case-population, providing a robust baseline against which to evaluate the impact of deprivation across the life-course.
- Analyses are constrained by the fact that national administrative healthcare data are primarily collected for administrative and clinical purposes, and not for research.
- With respect to weaknesses, lack of primary care data and information from psychosocial services are obvious deficiencies that currently affects all UK record-linkage studies of the type described here.

For peer review only - http://bmjopen.bmj.com/site/about/guidelines.xhtml

INTRODUCTION

In recent decades, advances in molecular biology and therapy have transformed the landscape for several childhood cancers, changing many from rapidly fatal diseases to treatable conditions with a good prognosis; overall survival now exceeds 80% in the UK and other economically developed countries[1,2]. Accordingly, it is estimated that by 2030 there will be around three-quarters of a million childhood cancer survivors in Europe alone; this number increasing as therapies improve and populations age[3]. Whilst the majority of cancers in children are more responsive to chemotherapy than those in adults, treatments are often more aggressive, and adverse health problems are well-known to occur later in life (e.g. second cancers, cardiac and bone problems, and fertility issues)[4–7].

With a view to investigating childhood cancer survivors' health, the national matched-cohort described in this report was established with the aim of examining the relationship between cancers diagnosed before 15 years of age and other morbidities and health-states in the years after cancer diagnosis; specific aims including the examination of secondary care hospital activity patterns, and the impact of deprivation on longer-term outcomes. The cohorts are predicated on the structures established in the United Kingdom Childhood Cancer Study (UKCCS), which was set-up as a national population-based case-control study in the early 1990s in England, Scotland and Wales to investigate a wide range of possible causes of childhood cancer. Collecting data from multiple sources (including interviews with parents, primary care records, obstetric/neonatal notes, birth certificates, household radiation measurements, and pre-treatment and remission blood samples), the case-control study investigated the potentially carcinogenic effects of a wide-range of physical (e.g. non-ionizing radiation), chemical (e.g. drugs) and biological (e.g. infectious agents) agents [8–14]. Examining associations in the pre-natal, in-utero, and post-natal periods, as well as associations with birth characteristics (e.g. birthweight) and other illnesses (e.g. allergies), over 90 reports have thus far been published (www.UKCCS.org).

Underpinned by updated ethics and legal permissions, the UKCCS has now been transformed into a matched population-based cohort, with follow-up linkages to electronic national administrative healthcare databases (inpatient and outpatient hospital episode statistics – HES, cancers, and deaths) [15–17]. Importantly, all cases and controls (regardless of parents' participation in the original case-control study) are being tracked forwards in time via annually updated linkages; the comparison cohort (controls) enabling robust baseline effect measures to be estimated. Currently over 25 years of follow-up data are available with which to investigate health-care patterns and health events occurring in cancer survivors. The children in the original UKCCS are presently aged between 27-46 years, and this report describes the study's underpinning cohort methods, and summarises some of the initial findings.

COHORT DESCRIPTION

Full details of the UKCCS case-control study (ukccs.org) which provides the foundation of the matched-cohort described here have been published[8,10,18]. Briefly, the sampling frame for cases and controls comprised all children registered with the National Health Service (NHS) in England, Scotland or Wales at the time the study was conducted (>98% of the childhood population in these countries); unfortunately for logistical reasons Northern Ireland (~3.5% of the total UK 0-14 population) was not included.

Overseen by a management committee that included epidemiologists, statisticians, paediatric oncologist/haematologists and expert scientists that was chaired by Professor Sir Richard Doll, 10

For peer review only - http://bmjopen.bmj.com/site/about/guidelines.xhtml

UKCCS administrative areas covering the whole of Britain were created for the purposes of data collection, with each being the responsibility of a well-established epidemiological research centre[8] (**Supplementary Figure 1**).

The study began on 1 January 1991 in Scotland, and either 1 April 1992 or 1 September 1992 in the nine UKCCS administrative areas of England and Wales. In Scotland, case accrual ended on 31 December 1994, and in England and Wales, it was restricted to children diagnosed with leukaemia or non-Hodgkin lymphoma throughout 1995, and leukaemia alone throughout 1996. In order to ensure ascertainment completeness, proactive cancer notification systems were established in all diagnostic/treatment centres across the UK; the majority of cases being notified at the point of diagnosis directly to the relevant UKCCS administrative centre by paediatric oncologists or haematologists practising in the study area. Subsequent crosschecks were made against regional childhood cancer registries (where they existed); the population-based National Registry of Childhood Tumours (NRCT), which covered England, Scotland and Wales [8,19]; and clinical trial datasets (acute lymphoblastic leukaemia).

With the permission of the child's clinical team, the parents of children with cancer were subsequently contacted. Each child whose parents agreed to be interviewed (3835/4430; 87%) was individually matched on sex, date of birth (month and year) and UKCCS region of residence to 10 controls that were randomly selected from the same population-based health service authority list as the case. With their general practitioner's (GP's) permission, the parents of the first two controls on the list ("first-choice" controls) were contacted and asked to participate in the study; but if the GP declined, or the parents did not wish to be interviewed, the next child was selected, and this procedure was repeated until two control families agreed to take part. By the end of the data collection phase, 96.7% (3786/3835) cases had two participating controls and 1.3% (49/3835) had one.

In order to monitor the characteristics of respondents and non-respondents across the study, and examine the potential impact of selection/participation bias, the registration details of all cases and controls were retained, regardless of whether or not their families were interviewed. Using standard methods, address postcodes were used to allocate deprivation scores for the home at diagnosis (all registered cases) or pseudo-diagnosis (all registered controls; date coinciding with the exact age that the corresponding case was diagnosed); and also at the time they were born (using mother's address on the child's birth certificate). Full details of the methods used have been previously published [8,18]. Briefly, deprivation categories were then derived by dividing the continuous score for the national 1991 census enumeration areas into five equally-sized groups, with group one representing the most affluent and group five the most deprived.

Illustrating the UKCCS's potential to examine issues relating to selection bias, the first two columns of **Table 1** distribute all registered subjects in the case-control study according to the area-based deprivation score of their home at diagnosis (cases) or pseudo-diagnosis (controls; date coinciding with the exact age that the corresponding case was diagnosed). With roughly 20% of the population in each quintile, the deprivation distribution of the 5-year survivor cohorts (last two columns of **Table 1,** right-side) mirror those of the case-control study (**Table 1,** left-side).

For peer review only - http://bmjopen.bmj.com/site/about/guidelines.xhtml

Table 1: Deprivation at cancer diagnosis/pseudo-diagnosis of subjects identified for inclusion on the case-control study and those for entry into the matched-cohorts

	Case-control-study: Subjects targeted for inclusion		Five-year Survivor cohorts	
	Cases	First-choice controls[1]	Cases	First choice controls
	N (%)	N (%)	N (%)	N (%)
**Deprivation**	4430 (100)	7663 (100)	3125 (100)	5611 (100)
**Affluent 1**	852 (19.2)	1501(19.6)	613(19.6)	1094(19.5)
**2**	913 (20.6)	1494 (19.5)	666(21.3)	1113(19.8)
**3**	852 (19.2)	1513 (19.7)	620(19.8)	1080(19.2)
**4**	891 (20.1)	1514 (19.8)	604(19.3)	1141(20.3)
**Deprived 5**	879 (19.8)	1587 (20.7)	604(19.3)	1140(20.3)
**Not known**	43 (1.0)	54 (0.7)	18(0.6)	43(0.8)
	χ^2=6.00, p=0.31		χ^2=5.57, p=0.35	

[1]Controls were not selected for 595 cases (13.4%) whose parent(s) were not interviewed; and only one suitable first-choice control was identified for 7 of the remaining 3835 cases.

As expected, the deprivation distribution of those whose parents participated in the case-control study differed from those who did not (**Table 2**). With 32.7% of non-participating controls residing in the most deprived quintile, the effect was more pronounced than in the case population (28.4%). Exacerbated by the fact that participation among case families was higher (3835/4430=86.6%) than among first-choice control families (5526/7663=72.1%), the deprivation distributions of interviewed cases (n=3835, column 1) and first-choice controls (n= 5526, column 3) differ significantly from each other (χ^2=15.1, p=0.004). As expected, distributing individuals in the 5-year survivors cohort by the same categories produced similar differences (**Table 2,** right-side). Such categorization, will not only allow us to evaluate the potential impact of deprivation at birth and diagnosis on hospital activity among 5-year survivors, but also to examine selection bias.

For peer review only - http://bmjopen.bmj.com/site/about/guidelines.xhtml

Table 2: Deprivation at cancer diagnosis/pseudo-diagnosis of subjects identified for inclusion on the case-control study and those for entry into the matched-cohorts distrivuted according to whether the parents participated in the case-control study or not.

	Case-control-study: Subjects targeted for inclusion				Five-year Survivor cohorts			
	Cases		First-choice Controls[1]		Cases		First-choice Controls	
	Parents participated?		Parents participated?		Parents participated?		Parents participated?	
	Yes	No	Yes	No	Yes	No	Yes	No
	N (%)	N (%)	N (%)	N (%)	N (%)	N (%)	N (%)	N (%)
**Total**	3835(100)	595(100)	5526(100)	2137(100)	2819(100)	306(100)	4080(100)	1531(100)
**Deprivation**								
**Affluent 1**	774(20.2)	78(13.1)	1249(22.6)	252(11.8)	576(20.4)	37(12.1)	915(22.4)	179(11.7)
**2**	823(21.5)	90(15.1)	1181(21.4)	313(14.7)	618(21.9)	48(15.7)	873(21.4)	240(15.7)
**3**	773(20.2)	79(13.3)	1156(20.9)	357(16.7)	579(20.5)	41(13.4)	839(20.6)	241(15.7)
**4**	755(19.7)	136(22.9)	1051(19.0)	463(21.7)	531(18.8)	73(23.9)	798(19.6)	343(22.4)
**Deprived 5**	710(18.5)	169(28.4)	889(16.1)	698(32.7)	515(18.3)	89(29.1)	655(16.0)	485(31.7)
**Not known**	-	43(7.2)	-	54(2.5)	-	18(5.9)	-	43(0.8)
	χ^2=64.8, p<0.001		χ^2=354.5, p<0.001		χ^2=43.3, p<0.001		χ^2=238.0, p<0.001	

[1]Controls were not selected for 595 cases (13.4%) whose parent(s) were not interviewed; and only one first-choice control was identified for 7 of the remaining 3835 cases.

Patient and public involvement and Engagement (PPIE)

The original case-control study was established over 30 years ago, at a time when lay-involvement in research was largely absent, and so did not benefit from formal links with patient/user groups. Members of the CCLG (Children's Cancer and Leukaemia Group, previously the UK Children's Cancer Study Group) were, however, fully involved throughout; and the study website (www.UKCCS.org) contains information about ongoing research activities and fair processing. The transformation of the study to focus on late effects among long-term childhood cancer survivors means that we can now move forwards to enable patients and the public to work in partnership with researchers and clinical staff to co-develop and deliver the research, disseminate findings, and determine the direction of future embedded studies. This input will ensure data are utilized in a way that secures maximize impact, improves long-term care, and promotes effective resource planning and management in the future.

Follow-up

The fact that basic details (matching variables, postcode, NHS number) of all cases and controls were registered and retained, regardless of the families' participation, enabled the conversion of the study into a matched cohort that could track all registered cases (n=4430) and their corresponding first-choice controls (n=7663) who were targeted for interview (**Table 1**; first two columns). In addition to the targeted case- and control-populations, replacement controls are being tracked, enabling the study to also make comparisons between the 3835 cases (**Table 2**, column 1) and 7621 controls (5526 first-choice (**Table 2**, column 3) and 2095 replacement controls) whose parents participated.

Tracking all of registered cases and controls, the UKCCS now operates on a legal basis that permits follow-up information to be obtained from NHS administrative healthcare records without explicit

For peer review only

For peer review only - http://bmjopen.bmj.com/site/about/guidelines.xhtml

consent, enabling all cases and their corresponding controls to be "flagged" at the national level and tracked via linkage to nationwide information on deaths, cancer registrations, and hospital episode data (inpatient day and overnight admissions; outpatient appointments and visits; accident and emergency presentations; and maternity admissions). Importantly, in line with current ethical and governance requirements, the matched-cohorts are now pseudonymized; NHS identifiers are no longer held by the study but by the national administrative bodies supplying linked data (NHS England, Public Health Scotland, NHS Central Register Scotland, and the Secured Anonymised Information Linkage Databank).

Beginning with the numbers registered in the original case-control study, the flow diagram presented in **Figure 1** gives the numbers of cases and controls who survived five years or more, followed by the numbers who were successfully linked to national administrative data sources**.** Cases enter the survivors' cohort exactly five years after their cancer was first diagnosed, and controls five years after their corresponding case's cancer was first diagnosed (pseudo-diagnosis date). Controls who emigrated, died or were lost to follow-up before five years had elapsed are not included in the survivor cohort, nor are controls who were matched to cases who died within five years of their diagnosis; since, where applicable, their follow-up was truncated when their matched case died (**Figure 1**; bottom row). All those who were targeted for interview in the original case-control study are shown on the left-side of **Figure 1**, and all those whose parents were interviewed on the right-side; the total number of individuals that are being followed is shown in the yellow boxes in the centre of the figure.

Figure 1

With respect to the available data, the time periods currently covered by the various linked healthcare datasets, which are updated on an annual basis are shown in **Supplementary Figure 2;** and the key data domains and fields are listed in **Box 1.**

Box 1: National administrative data available for comparative analysis: United Kingdom Childhood Cancer Study (UKCCS)

For peer review only - http://bmjopen.bmj.com/site/about/guidelines.xhtml

Source of data		Description of fields
Socio-demographic characteristics		Sex; year of birth; date of diagnosis/pseudo-diagnosis; indicators of deprivation at diagnosis/pseudo-diagnosis (Postcode sources; birth certificates, FHSA/HB[a] registers and parental interviews, HES[b])
Death notifications		Date and causes of death (ICD-10[c])
Emigrations		Date of embarkation
Cancer Registrations		Date of diagnosis; date of registration; topography (ICD revisions 7-10); morphology (ICD-O[d] revisions 1-3)
Secondary Care Data (Hospital Activity – HES[b]) Data		
	Inpatient and Day Cases	Date of admission; date of discharge; dates and types of procedures (OPCS-4[e], maximum 24); conditions at discharge (ICD-10[c], maximum 20); consultant specialties involved; source of referral; discharge destination
	Outpatient	Date of appointment and attendance flag; dates of procedures (maximum 24); consultant specialties involved
	Accident & Emergency	Date and reason for attendance; investigations and treatments; source of referral
	Maternity Inpatient and Day Cases	Date of admission to obstetrics or midwifery; date of discharge; conditions at discharge (ICD-10[c] maximum 20); age at birth; delivery method; babies' birthweights

[a]FHSA/HB: Family Health Strategic Authority or Health Board
[b]HES: Hospital Episode Statistics in England, or equivalently, Scottish Morbidity Record (SMR) in Scotland, and the Patient Episode Database for Wales (PEDW).
[c]ICD: International Classification of Diseases.
[d]ICD-O: International Classification of Diseases for Oncology.
[e]OPCS-4: Office of Population Censuses and Surveys Classification of Interventions and Procedures version 4.

For peer review only

Characteristics of individuals in the study

Detailed diagnostic and treatment information was obtained from multiple sources, including diagnosing/treatment centres, national treatment trials (where they existed), hospital records and reference laboratories. The diagnostic distribution, coded to the International Classification of Childhood Cancer 3rd edition (ICCC-3), as well as information on age, sex and interview status, is shown in **Table 3**. As expected, given the increasing emphasis on leukaemia in the later years of the case-control study's data collection phase, the final diagnostic distribution was weighted towards leukaemia. Variations with parental interview status are also evident; the non-interviewed group containing proportionally more CNS tumours (ICCC III) and other cancers (ICCC XI and XII). Among all registered cases, 1195 (27.0%) died within the first five years, and while in total, over 70% of the cases were alive at five years after diagnosis, the proportions within each ICCC-3 group varied with cancer (**Table 4**).

Table 3: Diagnostic frequencies, median ages at diagnosis, and parental interview status of children (0-14 years) registered in the United Kingdom Childhood Cancer Study (UKCCS)

Diagnosis (ICCC group)	Total N (%)	Males (%)	Age, median (interquartile range)	Interviewed Yes N (%)	Interviewed No N (%)
All cancers	4430(100.0)	55.7	5.1(2.7-9.7)	3835(100.0)	595(100.0)
Leukaemia (I)	1911(43.1)	55.8	4.5(2.8-8.0)	1734(45.2)	177(29.7)
Acute lymphoblastic leukaemia (Ia)	1580(35.7)	55.8	4.4(2.9-7.6)	1462(38.1)	118(19.8)
Acute myeloid leukaemia (Ib)	294(6.6)	53.7	5.4(1.8-11.2)	248(6.5)	46(7.7)
Lymphomas & reticuloendothelial (II)	413(9.3)	72.9	9.9(5.8-12.9)	357(9.3)	56(9.4)
Hodgkin lymphoma (IIa)	132(3.0)	71.2	11.6(7.9-13.5)	117(3.0)	15(2.5)
Non-Hodgkin lymphoma (IIb)	258(5.8)	72.9	9.4(5.2-12.0)	226(5.9)	32(5.4)
CNS, intracranial & intraspinal (III)	848(19.1)	50.1	6.5(3.3-10.1)	686(17.9)	162(27.2)
Ependymoma and choroid plexus (IIIa)	101(2.3)	58.4	3.4(1.4-9.0)	90(2.3)	11(1.8)
Astrocytoma (IIIb)	372(8.4)	44.1	6.9(4.0-10.5)	303(7.9)	69(11.6)
Intracranial & intraspinal embryonal (IIIc)	189(4.3)	55.6	6.0(2.7-8.8)	162(4.2)	27(4.5)
Neuroblastoma & other peripheral neural (IV)	222(5.0)	59.5	1.8(0.7-3.7)	188(4.9)	34(5.7)
Retinoblastoma (V)	112(2.5)	55.4	1.4(0.7-2.7)	87(2.3)	25(4.2)
Renal (VI)	221(5.0)	51.6	3.2(1.8-5.2)	199(5.2)	22(3.7)
Hepatic (VII)	36(0.8)	58.3	1.5(0.9-3.3)	32(0.8)	4(0.7)
Bone (VIII)	121(2.7)	53.7	11.2(8.9-13.5)	106(2.8)	15(2.5)
Soft tissue & other sarcomas (IX)	266(6.0)	54.9	5.7(2.8-10.6)	233(6.1)	33(5.5)
Germ cell & gonadal (X)	114(2.6)	54.4	6.9(1.4-12.3)	98(2.5)	16(2.7)
Other cancers (XI and XII)	99(2.2)	43.4	10.6(6.6-13.5)	62(1.6)	37(6.2)

For peer review only - http://bmjopen.bmj.com/site/about/guidelines.xhtml
For peer review only

Table 4: Number of children in the case-control study, numbers alive five years after diagnosis, and current duration of follow-up (July 2020 England and Wales; June 2022 Scotland) by diagnosis (ICCC-3): UKCCS

Diagnosis (ICCC group)	Total	Alive 5-years after diagnosis[1]		Total Person-Years[2]	
		N	%	Median (IQR)	Total
**Total Cases**	4430	3212	72.5	25.3(3.5-27.1)	78,623
Leukaemias (I)	1911	1448	75.8	24.7(5.2-26.7)	34,423
Acute lymphoblastic leukaemia (Ia)	1580	1275	80.7	24.9(9.0-26.9)	30,206
Acute myeloid leukaemia (Ib)	294	161	54.8	19.4(0.9-25.8)	3,913
Lymphomas & reticuloendothelial (II)	413	344	83.3	26.1(20.4-27.5)	8,434
Hodgkin lymphoma (IIa)	132	127	96.2	27.3(26.1-28.2)	3,194
Non-Hodgkin lymphoma (IIb)	258	203	78.7	25.4(12.9-27.1)	4,880
CNS, intracranial and intraspinal (III)	848	534	63.0	23.3(1.3-26.9)	13,021
Ependymoma and choroid plexus (IIIa)	101	62	61.4	9.2(1.9-26.5)	1,359
Astrocytoma (IIIb)	372	275	73.9	26.2(3.6-27.5)	6,894
Intracranial embryonal (IIIc)	189	84	44.4	3.0(0.8-26.1)	2,050
Neuroblastoma and other peripheral neural (IV)	222	121	54.5	14.7(1.3-26.8)	3,109
Retinoblastoma (V)	112	108	96.4	26.8(26.0-27.5)	2,769
Renal (VI)	221	173	78.3	26.2(19.4-27.4)	4,477
Hepatic (VII)	36	27	75.0	25.8(2.6-26.9)	674
Bone (VIII)	121	70	57.9	20.0(1.9-26.9)	1,799
Soft tissue and other sarcomas (IX)	266	167	62.8	25.2(1.8-27.4)	4,205
Germ cell and gonadal (X)	114	92	80.7	26.5(18.7-27.5)	2,359
Other cancers (XI & XII)	99	70	70.7	26.5(2.4-27.7)	1,793
**Total Controls**	9758	9703	99.4	26.5(25.2-27.6)	244,061
All first choice	7663	7601	99.2	26.6(25.2-27.6)	191,484
Replacements (parents interviewed)	2095	2091	99.8	26.5(25.1-27.5)	52,577

[1]97.7% of cases and 97.1% of controls have been traced and have administrative data.
[2]Person-years are calculated for the total number of cases from diagnosis to end of follow-up, and for controls from their pseudo-diagnosis date, i.e. the date they were the same age as their matched case was at diagnosis

FINDINGS TO DATE

The UKCCS provides a valuable resource within which to examine both the potential causes and long-term health consequences of childhood cancer. Thus far, over 90 peer-reviewed articles containing UKCCS data have been published, and a full list is on the study website (www.UKCCS.org). Assembling data from multiple sources, key reports include ALL genome-wide association studies (GWAS)[14,20], and those examining the potential aetiological roles of a wide range of biological (e.g. infections/markers of infectious exposure)[21–23], physical (e.g. ionizing/non-ionizing radiation)[12,13,24,25] and chemical (e.g. prescription drugs/smoking) agents acting on parents before the child's conception and the child during pregnancy/early life[10,26,27], as well as relationships with other illnesses (e.g. allergy) and birth characteristics (e.g. birthweight)[21,28,29]. Importantly, as noted above, the UKCCS accessed medical records to help answer key questions relating to prescription drugs, illness histories and co-morbidities; such contemporaneously recorded information, which by its nature is free from differential recall bias, often producing very different results from that obtained by self-report[11,22,30,31].

For peer review only - http://bmjopen.bmj.com/site/about/guidelines.xhtml

With respect to the longer-term health and healthcare needs of childhood cancer survivors, the conversion of the UKCCS into a matched cohort is now yielding important results about the morbidity and mortality of childhood cancer survivors. Early findings based on the case-population alone highlighted survival variations with age and sex across a range of subtypes[15], and for acute lymphoblastic leukaemia, survival associations with area-based measures of deprivation at the time of diagnosis were also noted[16]. With additional years of follow-up, we are now able to report a 25-year survival of 65.3% for all cancers combined (95% confidence interval 63.8-66.7). More recently, we have begun examining secondary care hospital activity patterns, comparing findings in the case-population to those in the control-population over the first 25 years. Adding to excess risks of death and cancer, our first report demonstrated that survivors of childhood acute lymphoblastic leukaemia (ALL) experienced excess outpatient and inpatient activity across their teenage and young adult years; which was not related to routine follow-up monitoring. Indeed, with hospital activity being higher than expected in specialties covering most organ and tissue systems, survivors were more than twice as likely to fall under the care of endocrinology, cardiology, respiratory medicine, ophthalmology, neurology and/or gastroenterology specialists[17]. Furthermore, these differences showed no signs of diminishing in the first 25 years of follow-up, underscoring the need to take prior cancer drug and/or radiation treatment into account when interpreting seemingly unrelated symptoms in later life.

STRENGTHS AND LIMITATIONS

Covering England, Scotland and Wales, findings from this population-based age- and sex- matched cohort study are based on routinely compiled linked health data, not self-report. With minimal selection bias and loss to follow-up,the matched-cohort design allows more granular analyses to be performed than is possible in case-cohorts that rely solely on general population rates, as has been done in other UK-based studies[32–35]. For example, at the point of cohort entry the individually age- and sex-matched control-population has a similar deprivation distribution to the case-population, providing a robust baseline against which to evaluate the potential impact of deprivation across the life-course. In addition, because the UKCCS cohorts can be divided into those whose families participated in the original case-control study and those who did not, the study is well-placed to investigate the impact of selection bias; an analysis which most cohorts cannot undertake, largely because they were not designed to do so, most being either record-based (as on the left side of **Figure 1**) [36–39]**,** or interview-based (as on the right-side of **Figure 1**) [40,41]. Thus far, with follow-up extending over 25 years from diagnosis, we have examined health through the TYA and early adult years, and indivduals in the matched-cohorts will continue to be tracked as they age. With respect to limitations, lack of reliable details about ethnicity, as well as information from primary care and psychosocial services are obvious deficiencies that currently affect all UK record-linkage studies of the type described here [42–44]. Analyses are also constrained by the fact that national administrative healthcare data are primarily collected for administrative and clinical purposes, and not for research.

COLLABORATION

To protect privacy and confidentiality, approval for the linkage of the UKCCS to health data is provided under strict conditions for the storage, retention and use of the data. The current approvals permit storage of the data at one site (University of York) for use by individually named researchers. We encourage interested parties to contact us to discuss potential analyses. Reasonable requests for data access can be submitted to the study Principal Investigator, ER (eve.roman@york.ac.uk), for review by the UKCCS investigator team. The PI will contact the relevant agencies to explore the feasibility of data sharing subject to ethical and data access agreements; should data sharing be agreeable with the agencies, approval for data access and

For peer review only - http://bmjopen.bmj.com/site/about/guidelines.xhtml

specific analyses will be required from the appropriate ethics committees and data sharing agreements put in place.

DATA SHARING

Reasonable requests for data sharing will be considered by the authors.

Ethics approval

The United Kingdom Childhood Cancer Study (UKCCS) has ethics approval from the Yorkshire & the Humber-Leeds West Research Ethics Committee (reference 18/YH/0135) and exemption from Section 251 of the Health & Social Care Act (reference 18/CAG/0066).

Author contributions

ER contributed to the design of the original case-control study, initiated the matched cohort, drafted the report, and is the guarantor. Contributing to both the original case-control and the matched cohort, SK and JS have also involved with the UKCCS since its inception. EK now leads on UKCCS data management, and was involved in the design of the matched cohort. AB, EK, JS, and AS have conducted many UKCCS analyses, and SK is the clinical lead for the study. DH and RS lead on PPIE, and have extensive experience of working with patients. All authors contributed to the written report.

Funding

The work reported on here was previously supported by Blood Cancer UK grant number 15037, and is jointly now supported by Blood Cancer UK and Cancer Research UK (grant number 29685). AB is supported by a fellowship from the Fondation ARC pour la Recherche sur le Cancer (PDF20190508759). ER and AS are supported in part by the National Institute for Health and Care Research (NIHR) Leeds Biomedical Research Centre. The funders had no role in considering the study design or in the collection, analysis, interpretation of data, writing of the manuscript, or the decision to submit the article for publication.

Conflicts of interest

None of the authors have any conflicts of interest

References

1 Gatta G, Botta L, Rossi S, *et al.* Childhood cancer survival in Europe 1999-2007: results of EUROCARE-5--a population-based study. *Lancet Oncol* 2014;**15**:35–47. doi:10.1016/S1470-2045(13)70548-5

2 American Cancer Society. Cancer Facts & Figures 2015. Atlanta, GA, USA: : American Cancer Society, Inc. 2015. https://www.cancer.org/research/cancer-facts-statistics/all-cancer-facts-figures/cancer-facts-figures-2015.html (accessed 2 Dec 2022).

3 The European Society for Paediatric Oncology. SIOP Europe Strategic Plan. Update (2021-2026). A European Cancer Plan for Children and Adolescents. Brussels, Belgium: : SIOP Europe 2021. https://siope.eu/european-strategic-plan/

4 Kebudi R, Özdemir GN. Second malignant neoplasms in childhood cancer survivors. *Curr Pediatr Rev* 2017;**13**:34–41.

5 Fidler MM, Reulen RC, Henson KE, *et al.* Population-Based Long-Term Cardiac-Specific Mortality Among 34,489 Five-Year Survivors of Childhood Cancer in Great Britain. *Circulation* 2017;**135**:951–63. doi:10.1161/CIRCULATIONAHA.116.024811

For peer review only - http://bmjopen.bmj.com/site/about/guidelines.xhtml

6 Dietz AC, Chen Y, Yasui Y, *et al.* Risk and impact of pulmonary complications in survivors of childhood cancer: A report from the Childhood Cancer Survivor Study. *Cancer* 2016;**122**:3687–96. doi:10.1002/cncr.30200

7 Overbeek A, van den Berg MH, van Leeuwen FE, *et al.* Chemotherapy-related late adverse effects on ovarian function in female survivors of childhood and young adult cancer: A systematic review. *Cancer Treat Rev* 2017;**53**:10–24. doi:10.1016/j.ctrv.2016.11.006

8 UK Childhood Cancer Study Investigators. The United Kingdom Childhood Cancer Study: objectives, materials and methods. *Br J Cancer* 2000;**82**:1073–102. doi:10.1054/bjoc.1999.1045

9 Day N, Skinner J, Roman E, *et al.* Exposure to power-frequency magnetic fields and the risk of childhood cancer. UK Childhood Cancer Study Investigators. *Lancet* 1999;**354**:1925–31.

10 Roman E, Simpson J, Ansell P, *et al.* Perinatal and reproductive factors: a report on haematological malignancies from the UKCCS. *Eur J Cancer* 2005;**41**:749–59. doi:10.1016/j.ejca.2004.11.006

11 Roman E, Simpson J, Ansell P, *et al.* Infectious proxies and childhood leukaemia: Findings from the United Kingdom Childhood Cancer Study (UKCCS). *Blood Cells, Molecules, and Diseases* 2009;**42**:126–8. doi:10.1016/j.bcmd.2008.10.007

12 UK Childhood Cancer Study Investigators. The United Kingdom Childhood Cancer Study of exposure to domestic sources of ionising radiation: 1: radon gas. *Br J Cancer* 2002;**86**:1721–6. doi:10.1038/sj.bjc.6600276

13 UK Childhood Cancer Study Investigators. The United Kingdom Childhood Cancer Study of exposure to domestic sources of ionising radiation: 2: gamma radiation. *Br J Cancer* 2002;**86**:1727–31. doi:10.1038/sj.bjc.6600277

14 Papaemmanuil E, Hosking FJ, Vijayakrishnan J, *et al.* Loci on 7p12.2, 10q21.2 and 14q11.2 are associated with risk of childhood acute lymphoblastic leukemia. *Nat Genet* 2009;**41**:1006–10. doi:10.1038/ng.430

15 Johnston WT, Lightfoot TJ, Simpson J, *et al.* Childhood cancer survival: a report from the United Kingdom Childhood Cancer Study. *Cancer Epidemiol* 2010;**34**:659–66. doi:10.1016/j.canep.2010.06.020

16 Lightfoot TJ, Johnston WT, Simpson J, *et al.* Survival from childhood acute lymphoblastic leukaemia: the impact of social inequality in the United Kingdom. *Eur J Cancer* 2012;**48**:263–9. doi:10.1016/j.ejca.2011.10.007

17 Kane E, Kinsey S, Bonaventure A, *et al.* Excess morbidity and mortality among survivors of childhood acute lymphoblastic leukaemia: 25 years of follow-up from the United Kingdom Childhood Cancer Study (UKCCS) population-based matched cohort. *BMJ Open* 2022;**12**:e056216. doi:10.1136/bmjopen-2021-056216

18 Smith A, Roman E, Simpson J, *et al.* Childhood leukaemia and socioeconomic status: fact or artefact? A report from the United Kingdom childhood cancer study (UKCCS). *International Journal of Epidemiology* 2006;**35**:1504–13. doi:10.1093/ije/dyl193

19 Stiller CA, Allen MB, Eatock EM. Childhood cancer in Britain: the National Registry of Childhood Tumours and incidence rates 1978-1987. *Eur J Cancer* 1995;**31A**:2028–34.

For peer review only

For peer review only - http://bmjopen.bmj.com/site/about/guidelines.xhtml

20 Vijayakrishnan J, Kumar R, Henrion MYR, *et al.* A genome-wide association study identifies risk loci for childhood acute lymphoblastic leukemia at 10q26.13 and 12q23.1. *Leukemia* 2017;**31**:573–9. doi:10.1038/leu.2016.271

21 Roman E, Simpson J, Ansell P, *et al.* Childhood Acute Lymphoblastic Leukemia and Infections in the First Year of Life: A Report from the United Kingdom Childhood Cancer Study. *American Journal of Epidemiology* 2007;**165**:496–504. doi:10.1093/aje/kwk039

22 Simpson J, Smith A, Ansell P, *et al.* Childhood leukaemia and infectious exposure: A report from the United Kingdom Childhood Cancer Study (UKCCS). *European Journal of Cancer* 2007;**43**:2396–403. doi:10.1016/j.ejca.2007.07.027

23 Crouch S, Lightfoot T, Simpson J, *et al.* Infectious illness in children subsequently diagnosed with acute lymphoblastic leukemia: modeling the trends from birth to diagnosis. *American journal of epidemiology* 2012;**176**:402–8.

24 Ahlbom A, Day N, Feychting M, *et al.* A pooled analysis of magnetic fields and childhood leukaemia. *Br J Cancer* 2000;**83**:692–8. doi:10.1054/bjoc.2000.1376

25 Skinner J, Mee TJ, Blackwell RP, *et al.* Exposure to power frequency electric fields and the risk of childhood cancer in the UK. *Br J Cancer* 2002;**87**:1257–66. doi:10.1038/sj.bjc.6600602

26 Roman E, Fear NT, Ansell P, *et al.* Vitamin K and childhood cancer: analysis of individual patient data from six case–control studies. *British Journal of Cancer* 2002;**86**:63–9.

27 Bonaventure A, Simpson J, Ansell P, *et al.* Prescription drug use during pregnancy and risk of childhood cancer – Is there an association? *Cancer Epidemiology* 2015;**39**:73–8. doi:10.1016/j.canep.2014.10.008

28 Bonaventure A, Simpson J, Ansell P, *et al.* Paediatric acute lymphoblastic leukaemia and caesarean section: A report from the United Kingdom Childhood Cancer Study (UKCCS). *Paediatric and Perinatal Epidemiology* 2020;**34**:344–9. doi:10.1111/ppe.12662

29 Milne E, Greenop KR, Metayer C, *et al.* Fetal growth and childhood acute lymphoblastic leukemia: Findings from the childhood leukemia international consortium. *International Journal of Cancer* 2013;**133**:2968–79. doi:10.1002/ijc.28314

30 Smith A, Roman E, Simpson J. Childhood leukaemia and socioeconomic status. *International Journal of Epidemiology* 2007;**36**:1156. doi:10.1093/ije/dym166

31 Bonaventure A, Kane E, Simpson J, *et al.* Maternal infections and medications in pregnancy: how does self-report compare to medical records in childhood cancer case–control studies? *International Journal of Epidemiology* 2023;:dyad019. doi:10.1093/ije/dyad019

32 van Laar M, Feltbower RG, Gale CP, *et al.* Cardiovascular sequelae in long-term survivors of young peoples' cancer: a linked cohort study. *Br J Cancer* 2014;**110**:1338–41. doi:10.1038/bjc.2014.37

33 Reulen RC, Bright CJ, Winter DL, *et al.* Pregnancy and Labor Complications in Female Survivors of Childhood Cancer: The British Childhood Cancer Survivor Study. *J Natl Cancer Inst* 2017;**109**. doi:10.1093/jnci/djx056

For peer review only

For peer review only - http://bmjopen.bmj.com/site/about/guidelines.xhtml

34 Smith L, Glaser AW, Peckham D, *et al.* Respiratory morbidity in young people surviving cancer: Population-based study of hospital admissions, treatment-related risk factors and subsequent mortality. *Int J Cancer* 2019;**145**:20–8. doi:10.1002/ijc.32066

35 Reulen RC, Guha J, Bright CJ, *et al.* Risk of cerebrovascular disease among 13 457 five-year survivors of childhood cancer: A population-based cohort study. *International Journal of Cancer* 2021;**148**:572–83. doi:10.1002/ijc.33218

36 McBride ML, Rogers PC, Sheps SB, *et al.* Childhood, adolescent, and young adult cancer survivors research program of British Columbia: objectives, study design, and cohort characteristics. *Pediatr Blood Cancer* 2010;**55**:324–30. doi:10.1002/pbc.22476

37 Asdahl PH, Winther JF, Bonnesen TG, *et al.* The Adult Life After Childhood Cancer in Scandinavia (ALiCCS) Study: Design and Characteristics. *Pediatr Blood Cancer* 2015;**62**:2204–10. doi:10.1002/pbc.25661

38 Kirchhoff AC, Fluchel MN, Wright J, *et al.* Risk of Hospitalization for Survivors of Childhood and Adolescent Cancer. *Cancer Epidemiol Biomarkers Prev* Published Online First: 12 June 2014. doi:10.1158/1055-9965.EPI-13-1090

39 Streefkerk N, Tissing WJE, Korevaar JC, *et al.* A detailed insight in the high risks of hospitalizations in long-term childhood cancer survivors-A Dutch LATER linkage study. *PLoS ONE* 2020;**15**:e0232708. doi:10.1371/journal.pone.0232708

40 Robison LL, Mertens AC, Boice JD, *et al.* Study design and cohort characteristics of the Childhood Cancer Survivor Study: a multi-institutional collaborative project. *Med Pediatr Oncol* 2002;**38**:229–39. doi:10.1002/mpo.1316

41 Hawkins MM, Lancashire ER, Winter DL, *et al.* The British Childhood Cancer Survivor Study: Objectives, methods, population structure, response rates and initial descriptive information. *Pediatr Blood Cancer* 2008;**50**:1018–25. doi:10.1002/pbc.21335

42 Chow EJ, Ness KK, Armstrong GT, *et al.* Current and coming challenges in the management of the survivorship population. *Semin Oncol* 2020;**47**:23–39. doi:10.1053/j.seminoncol.2020.02.007

43 Andrés-Jensen L, Skipper MT, Mielke Christensen K, *et al.* National, clinical cohort study of late effects among survivors of acute lymphoblastic leukaemia: the ALL-STAR study protocol. *BMJ Open* 2021;**11**. doi:10.1136/bmjopen-2020-045543

44 van Erp LME, Maurice-Stam H, Kremer LCM, *et al.* A vulnerable age group: the impact of cancer on the psychosocial well-being of young adult childhood cancer survivors. *Support Care Cancer* 2021;**29**:4751–61. doi:10.1007/s00520-021-06009-y

For peer review only - http://bmjopen.bmj.com/site/about/guidelines.xhtml

Figure Legends

Figure 1: UKCCS subjects distributed by parental interviewed status (total targeted and total interviewed) and follow-up status; the numbers of 5-year survivors with linked health data that are being followed are presented in the bottom row.

Supplementary Figure 1: United Kingdom Childhood Cancer Study (UKCCS) administrative areas

Supplementary Figure 2: United Kingdom Childhood Cancer Study (UKCCS) linkage to national administrative datas

For peer review only

For peer review only - http://bmjopen.bmj.com/site/about/guidelines.xhtml

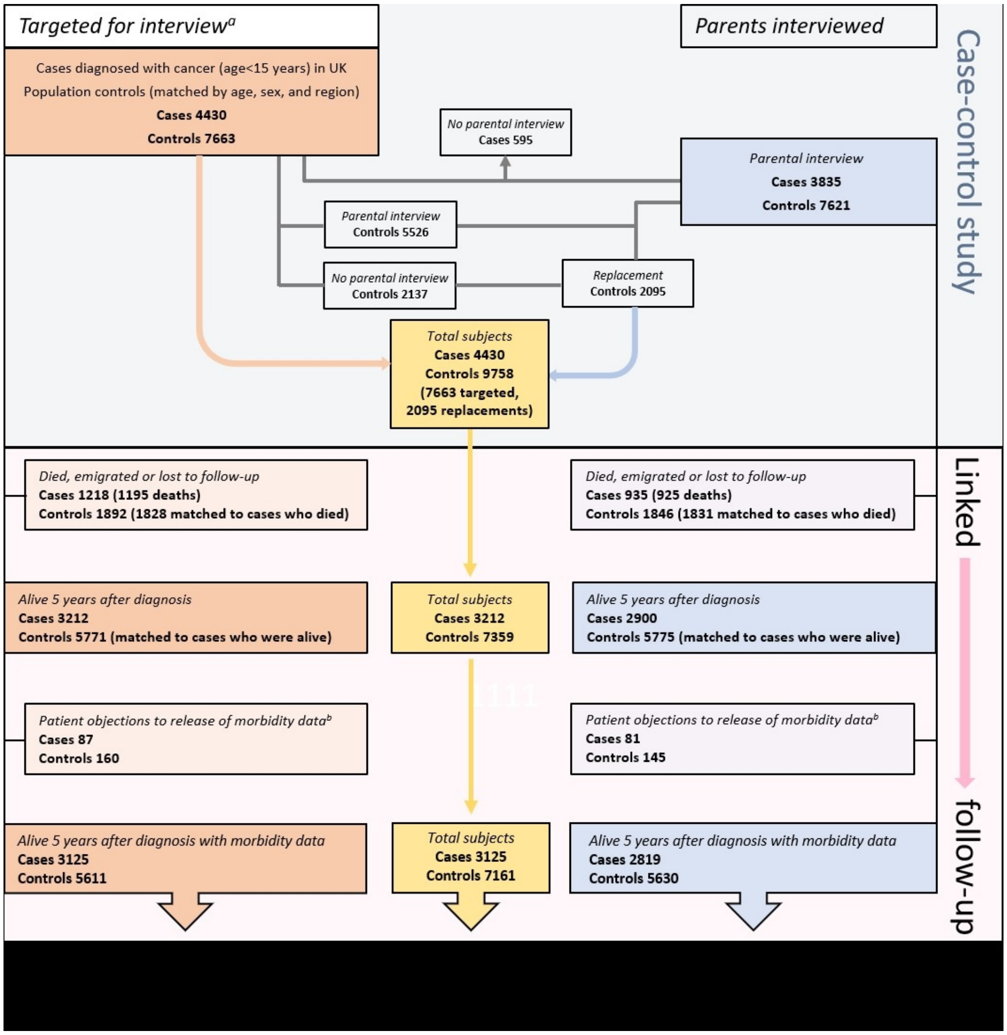

Figure 1: UKCCS subjects distributed by parental interviewed status (total targeted and total interviewed) and follow-up status; the numbers of 5-year survivors with linked health data that are being followed are presented in the bottom row.

180x184mm (150 x 150 DPI)

Supplementary Figure 1

United Kingdom Childhood Cancer Study (UKCCS) administrative areas

Study Population

- Children (≤ 15 years) registered for primary care with a general practitioner - > 98% of all children in UK

Cases - ascertained from multiple sources:-

- Treating/ referring hospitals
- Cross-checks with cancer registries subsequently confirming the completeness of ascertainment

Controls - 2 per case selected from primary care population registers, individually matched on:-

- Sex
- Year and month of birth
- UKCCS region of residence

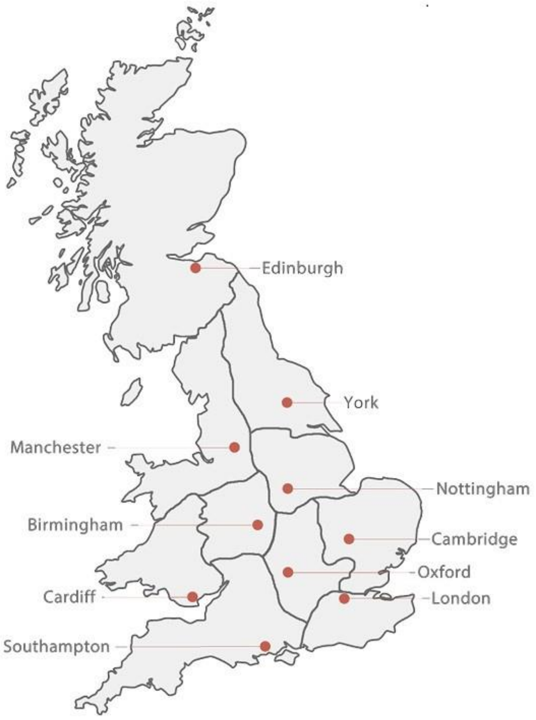

eview only

Supplementary Figure 2

United Kingdom Childhood Cancer Study (UKCCS) linkage to national administrative datasets

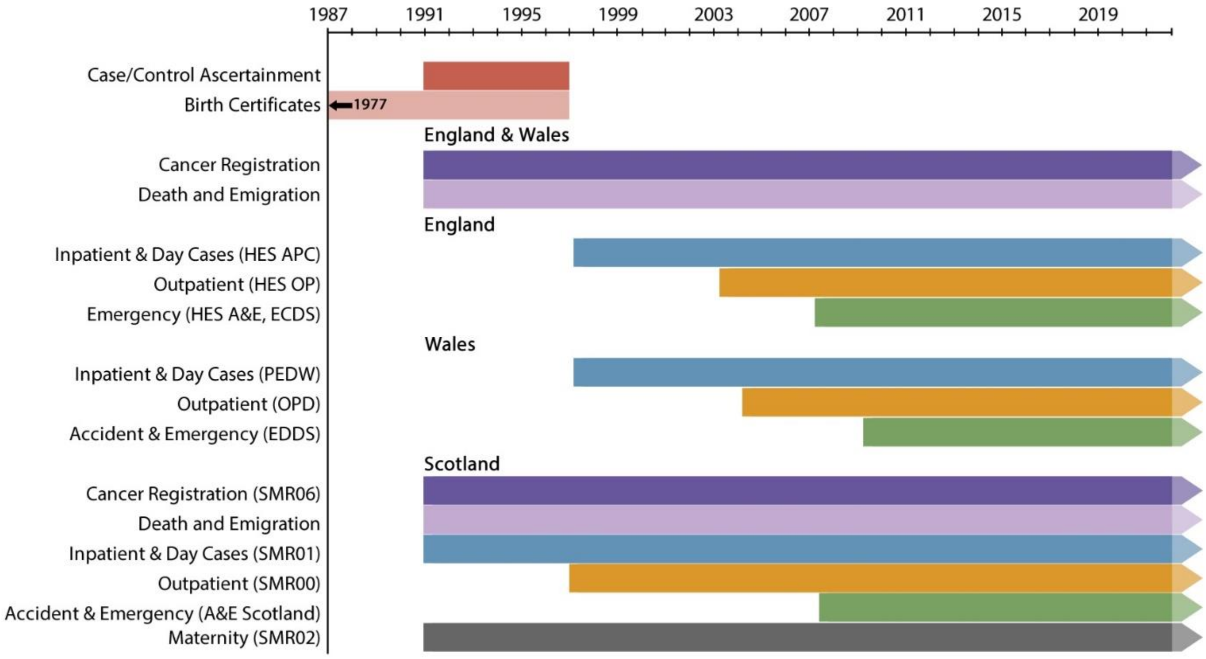

UKCCS: United Kingdom Childhood Cancer Study; HES: Hospital Episode Statistics; APC: Admitted Patient Care; OP: Outpatients; ECDS: Emergency Care Dataset; PEDW: Patient Episode Data for Wales; OPD: Outpatient Data (Wales); EDD: Emergency Department Dataset (Wales); SMR: Scottish Morbidity Record

For peer review only

STROBE 2007 (v4) Statement—Checklist of items that should be included in reports of *cohort studies*

Section/Topic	Item #	Recommendation	Reported on page #
**Title and abstract**	1	(*a*) Indicate the study's design with a commonly used term in the title or the abstract	1-2
		(*b*) Provide in the abstract an informative and balanced summary of what was done and what was found	2
**Introduction**			
Background/rationale	2	Explain the scientific background and rationale for the investigation being reported	2-4
Objectives	3	State specific objectives, including any prespecified hypotheses	2
**Methods**			
Study design	4	Present key elements of study design early in the paper	2
Setting	5	Describe the setting, locations, and relevant dates, including periods of recruitment, exposure, follow-up, and data collection	2-5
Participants	6	(*a*) Give the eligibility criteria, and the sources and methods of selection of participants. Describe methods of follow-up	3-4
		(*b*) For matched studies, give matching criteria and number of exposed and unexposed	3
Variables	7	Clearly define all outcomes, exposures, predictors, potential confounders, and effect modifiers. Give diagnostic criteria, if applicable	3-6
Data sources/ measurement	8*	For each variable of interest, give sources of data and details of methods of assessment (measurement). Describe comparability of assessment methods if there is more than one group	3-6
Bias	9	Describe any efforts to address potential sources of bias	3-6
Study size	10	Explain how the study size was arrived at	3-8
Quantitative variables	11	Explain how quantitative variables were handled in the analyses. If applicable, describe which groupings were chosen and why	3-7
Statistical methods	12	(*a*) Describe all statistical methods, including those used to control for confounding	3-6
		(*b*) Describe any methods used to examine subgroups and interactions	5
		(*c*) Explain how missing data were addressed	5
		(*d*) If applicable, explain how loss to follow-up was addressed	5
		(*e*) Describe any sensitivity analyses	NA
**Results**			

For peer review only

Participants	13*	(a) Report numbers of individuals at each stage of study—eg numbers potentially eligible, examined for eligibility, confirmed eligible, included in the study, completing follow-up, and analysed	5
		(b) Give reasons for non-participation at each stage	3-5
		(c) Consider use of a flow diagram	3
Descriptive data	14*	(a) Give characteristics of study participants (eg demographic, clinical, social) and information on exposures and potential confounders	4,7,8
		(b) Indicate number of participants with missing data for each variable of interest	8
		(c) Summarise follow-up time (eg, average and total amount)	8
Outcome data	15*	Report numbers of outcome events or summary measures over time	8
Main results	16	(*a*) Give unadjusted estimates and, if applicable, confounder-adjusted estimates and their precision (eg, 95% confidence interval). Make clear which confounders were adjusted for and why they were included	NA
		(*b*) Report category boundaries when continuous variables were categorized	NA
		(*c*) If relevant, consider translating estimates of relative risk into absolute risk for a meaningful time period	NA
Other analyses	17	Report other analyses done—eg analyses of subgroups and interactions, and sensitivity analyses	NA
**Discussion**			
Key results	18	Summarise key results with reference to study objectives	8-9
**Limitations**			
Interpretation	20	Give a cautious overall interpretation of results considering objectives, limitations, multiplicity of analyses, results from similar studies, and other relevant evidence	8-9
Generalisability	21	Discuss the generalisability (external validity) of the study results	9
**Other information**			
Funding	22	Give the source of funding and the role of the funders for the present study and, if applicable, for the original study on which the present article is based	10

*Give information separately for cases and controls in case-control studies and, if applicable, for exposed and unexposed groups in cohort and cross-sectional studies.

Note: An Explanation and Elaboration article discusses each checklist item and gives methodological background and published examples of transparent reporting. The STROBE checklist is best used in conjunction with this article (freely available on the Web sites of PLoS Medicine at http://www.plosmedicine.org/, Annals of Internal Medicine at http://www.annals.org/, and Epidemiology at http://www.epidem.com/). Information on the STROBE Initiative is available at www.strobe-statement.org.

For peer review only
